# Comparison of the Informed Health Choices Key Concepts Framework to other frameworks relevant to teaching and learning how to think critically about health claims and choices: a systematic review

**DOI:** 10.12688/f1000research.21858.1

**Published:** 2020-03-05

**Authors:** Andrew D. Oxman, Laura Martínez García

**Affiliations:** 1 Centre for Informed Health Choices, Norwegian Institute of Public Health, Oslo, Norway; 2 University of Oslo, Oslo, Norway; 3 Iberoamerican Cochrane Centre, Sant Pau Biomedical Research Institute, Barcelona, Spain; 4 CIBER of Epidemiology and Public Health, Barcelona, Spain

**Keywords:** critical thinking, evidence-informed decision-making, evidence-based practice, evidence informed decision-making, argumentation, causal inference, cognitive biases, epistemic cognition, health literacy, logical fallacies, meta-cognition, scientific thinking, frameworks, models, concepts, competences, concepts

## Abstract

**Background:** The Informed Health Choices (IHC) Key Concepts are principles for evaluating the trustworthiness of claims about treatment effects. The Key Concepts provide a framework for developing learning-resources to help people use the concepts when treatment claims are made, and when they make health choices.

**Objective: **To compare the framework provided by the IHC Key Concepts to other frameworks intended to promote critical thinking about treatment (intervention) claims and choices.

**Methods:** We identified relevant frameworks from reviews of frameworks, searching Google Scholar, citation searches, and contact with key informants. We included frameworks intended to provide a structure for teaching or learning to think critically about the basis for claims, evidence used to support claims, or informed choices. For a framework to be included, there had to be a description of its purpose; a list of concepts, competences, or dispositions; and definitions of key terms. We made independent assessments of framework eligibility and extracted data for each included framework using standardised forms.

**Results: **Twenty-two frameworks met our inclusion criteria. The purpose of the IHC Framework is similar to that of two frameworks for critical thinking and somewhat similar to that of a framework for evidence-based practice. Those frameworks have broader scopes than the IHC Framework. An important limitation of broad frameworks is that they do not provide an adequate basis (concepts) for deciding which claims to believe and what to do. There was at most some overlap between the concepts, competences, and dispositions in each of the 22 included frameworks and those in the IHC Framework.

**Conclusions: **The IHC Key Concepts Framework appears to be unique.  Our review has shown how it and other frameworks can be improved by taking account of the ways in which other related frameworks have been developed, evaluated, and made useful.

## Introduction

### The Informed Health Choices (IHC) Key Concepts Framework

Claims about what people can do to improve or protect their health (treatments) are ubiquitous. They are found in the mass media, advertisements, and everyday personal communication. Some are based on trustworthy evidence. Many are not, and many people have difficulties determining which claims to believe and act on. Acting on untrustworthy claims and not acting on ones that are trustworthy can result in unnecessary suffering and waste.

In response to these challenges, we developed the Informed Health Choices (IHC) Key Concepts as the first step in the IHC project ^1– 4^. The aim of the IHC project is to help people, particularly primary and secondary school students, learn to assess treatment claims and make informed health choices ^5^.

We use ‘treatment’ to refer to any intervention or action intended to protect or improve health ^6^. People in other fields have found the IHC Key Concepts relevant for assessing claims about the effects of other types of interventions ^7^. This includes agricultural, educational, environmental, management, social welfare, economic, international development, nutrition, policing, and veterinary interventions.

The IHC Key Concepts provide a framework for designing curricula, learning resources, and evaluation tools ^5, 8^. We first published the framework in 2015 ^1^ and have continued to update it yearly. The current (2019) framework includes 49 concepts in three groups ( Table 1), 20 competences in four groups ( Table 2), and 16 dispositions in four groups ( Table 3) ^4^. The concepts are principles for evaluating the trustworthiness of treatment claims and the evidence used to support these, and for making informed choices. The methods used to develop the framework are described elsewhere ^1, 3^. The framework is a starting point to help teachers, journalists, researchers and other intermediaries to identify and develop resources to help people learn to assess treatment claims and make informed choices.

**Table 1.** Overview of the IHC Key Concepts.

1. Claims *Claims about effects that are not supported by* *evidence from fair comparisons are not necessarily* *wrong, but there is an insufficient basis for* *believing them.*	2. Comparisons *Studies should make fair comparisons, designed* *to minimize the risk of systematic errors (biases)* *and random errors (the play of chance).*	3. Choices *What to do depends on judgements* *about a problem, the relevance* *of the evidence available, and* *the balance of expected benefits,* *harms, and costs.*
**1.1 It should not be assumed that treatments are** **safe or effective - or that they are not.** a) Treatments can cause harms as well as benefits. b) Large, dramatic effects are rare. c) It is rarely possible to be certain about the effects of treatments. **1.2 Seemingly logical assumptions are not a** **sufficient basis for claims.** a) Treatment may not be needed. b) Beliefs alone about how treatments work are not reliable predictors of the presence or size of effects. c) Assumptions that fair comparisons of treatments in research are not applicable in practice can be misleading. d) An outcome may be associated with a treatment but not caused by it. e) More data is not necessarily better data. f) Identifying effects of treatments depends on making comparisons. g) The results of one study considered in isolation can be misleading. h) Widely used treatments or those that have been used for decades are not necessarily beneficial or safe. i) Treatments that are new or technologically impressive may not be better than available alternatives. j) Increasing the amount of a treatment does not necessarily increase its benefits and may cause harm. k) Earlier detection of ‘disease’ is not necessarily better. l) It is rarely possible to know in advance who will benefit, who will not, and who will be harmed by using a treatment. **1.3 Trust in a source alone is not a sufficient basis** **for believing a claim.** a) Your existing beliefs may be wrong. b) Competing interests may result in misleading claims. c) Personal experiences or anecdotes alone are an unreliable basis for most claims. d) Opinions alone are not a reliable basis for claims. e) Peer review and publication by a journal do not guarantee that comparisons have been fair.	**2.1 Comparisons of treatments should be fair.** a) Comparison groups should be as similar as possible. b) Indirect comparisons of treatments across different studies can be misleading. c) The people being compared should be cared for similarly apart from the treatments being studied. d) If possible, people should not know which of the treatments being compared they are receiving. e) Outcomes should be assessed in the same way in all the groups being compared. f) Outcomes should be assessed using methods that have been shown to be reliable. g) It is important to assess outcomes in all (or nearly all) the people in a study. h) People’s outcomes should be counted in the group to which they were allocated **.** **2.2 Syntheses of studies need to be reliable.** a) Reviews of studies comparing treatments should use systematic methods. b) Failure to consider unpublished results of fair comparisons may result in estimates of effects that are misleading. c) Treatment claims based on models may be sensitive to underlying assumptions. **2.3 Descriptions should clearly reflect the size** **of effects and the risk of being misled by the** **play of chance.** a) Verbal descriptions of the size of effects alone can be misleading. b) Relative effects of treatments alone can be misleading. c) Average differences between treatments can be misleading. d) Small studies may be misleading. e) Results for a selected group of people within a study can be misleading. f) The use of p-values may be misleading; confidence intervals are more informative. g) Deeming results to be “statistically significant” or “nonsignificant” can be misleading. h) Lack of evidence of a difference is not the same as evidence of “no difference”.	**3.1 Problems and options should** **be clear.** a) Be clear about what the problem or goal is and what the options are. **3.2 Evidence should be relevant.** a) Attention should focus on all important effects of treatments, and not surrogate outcomes. b) Fair comparisons of treatments in animals or highly selected groups of people may not be relevant. c) The treatments compared should be similar to those of interest. d) There should not be important differences between the circumstances in which the treatments were compared and those of interest. **3.3 Expected advantages should** **outweigh expected disadvantages.** a) Weigh the benefits and savings against the harms and costs of acting or not. b) Consider the baseline risk or the severity of the symptoms when estimating the size of expected effects. c) Consider how important each advantage and disadvantage is when weighing the pros and cons. d) Consider how certain you can be about each advantage and disadvantage. e) Important uncertainties about the effects of treatments should be addressed in further fair comparisons.

**Table 2.** IHC competences.

**Goal** To enable people to make good decisions * about which claims to believe about the effects of things they can do for their health, the health of others or for other reasons, and about what to do to achieve their goals. **Competences** To achieve this goal, people should be able to: **1.** **Recognise when a claim has an untrustworthy basis by:** a) recognising claims about the effects of treatments b) questioning the basis for treatment claims c) thinking carefully about treatment claims before believing them d) recognising when a treatment claim is relevant and important, and warrants reflection **2.** **Recognise when evidence used to support a treatment claim is trustworthy or untrustworthy by:** a) recognising the assumptions, evidence and reasoning behind treatment claims b) recognising unfair treatment comparisons c) recognising unreliable summaries of treatment comparisons d) recognising when a statistical model and its assumptions are used to support a treatment claim e) recognising misleading ways of presenting treatment effects f) understanding how systematic errors (the risk of bias), random errors (the play of chance), and the relevance (applicability) of treatment comparisons can affect the degree of confidence in estimates of treatment effects g) understanding the extent to which evidence does or does not support a treatment claim **3.** **Make well-informed decisions about treatments by:** a) being aware of cognitive biases when making decisions b) clarifying and understanding the problem, options, and goals when making a decision c) recognising when decisions have irreversible consequences d) judging the relevance of evidence used to inform decisions about treatments e) weighing the advantages and disadvantages of treatments, taking into account the size of treatment effects, how important each outcome is, the costs, and the certainty of the evidence f) communicating with others about the advantages and disadvantages of treatments **4.** **Reflect on people’s competences and dispositions by:** a) monitoring how they decide which treatment claims to believe and what to do b) monitoring how people adjust the processes they use to decide what to believe and do to fit the relevance, importance, and nature of different types of treatment claims and choices c) being aware of when people are making treatment claims themselves

*A good decision is one that makes effective use of the information available to the decision maker at the time the decision is made. A good outcome is one that the decision maker likes. The aim of thinking critically about treatments is to increase the probability of good outcomes (and true conclusions), but many other factors affect outcomes aside from critical thinking ^36^.

**Table 3.** IHC dispositions.

People should be in the habit of thinking critically about: **1.** **Claims by:** a) being aware of treatment claims (including those you make yourself) and choices b) questioning the basis for treatment claims c) being aware of cognitive biases and going from fast to slow thinking before forming an opinion about a treatment claim, making a claim, or taking a decision d) seeking evidence to reduce uncertainty when considering a relevant and important treatment claim or decision **2.** **Evidence used to support claims by:** a) questioning the trustworthiness of evidence used to support treatment claims b) being alert to misleading presentations of treatment effects c) acknowledging and accepting uncertainty about the effects of treatments d) being willing to admit errors and modify their judgements when warranted by evidence or a lack of evidence **3.** **Choices by:** a) clarifying and understanding the problem, options, and goals when making decisions about treatments b) preferring evidence-based sources of information about treatment effects c) considering the relevance of the evidence used to inform decisions about treatments d) considering effect estimates, baseline risk, the importance of each advantage and disadvantage, the costs, and the certainty of the evidence when making decisions about treatments e) making informed judgements about the certainty of estimates of treatment effects f) making well-informed decisions g) Being aware of how people decide which treatment claims to believe and what to do **4.** **People’s own thinking by:** a) Being aware of how people decide which treatment claims to believe and what to do

### Other frameworks relevant to the IHC Key Concepts Framework

There are many other frameworks that include concepts, competences, or dispositions that are relevant to thinking critically about treatment claims, comparisons, and choices. These include critical thinking frameworks, logical fallacies and argumentation frameworks, cognitive frameworks, frameworks for scientific thinking, and frameworks related to evidence-based health care. For each category of frameworks there are disagreements about definitions and what is included. For example, learning to think critically is widely held as an aim of education ^9^, but there is not agreement on the definition of “critical thinking” and there are several different frameworks (conceptual structures intended to serve as a support or guide) for critical thinking ^10– 14^. Similarly, there are different definitions and frameworks for scientific thinking (reasoning and literacy) ^15– 18^, epistemic cognition and meta-cognition ^19, 20^, health literacy ^21– 23^, and various aspects of evidence-based health care ^24– 26^. There is also overlap across these different framework categories, some of which have been grouped together as frameworks for “productive thinking” ^12^.

### Terminology

Definitions of terms that we use in this paper are shown in Table 4.

**Table 4.** Definitions of terms as used in this paper.

Choice	A decision to do something (or not to do something) with the intention of achieving a goal, such as improving or maintaining health
Claim	A statement about what will happen if one action (e.g. a treatment) is chosen compared to what would happen if another action (or “no treatment”) was chosen
Comparison	Examination of the evidence for differences between two options, such as what will happen if one action is chosen compared to what would happen if another action was chosen
Competency	The required skill, knowledge, or capacity to do something
Concept	In this review, concept (an idea, object of thought, or constituent of thought) refers to a specific type of concept: a criterion (standard for judgment) or principle (a concept that is a guide) for evaluating the trustworthiness of claims and comparisons, and for making choices; or an issue worthy of attention or consideration when assessing claims and making choices.
Curriculum	A set of learning goals that outline the intended content and process goals of a school program
Disposition	Frequent and voluntary habits of thinking and doing
Domain	A group of elements within a framework
Element	One of the components of a framework, including concepts, competences, and dispositions
Fair comparison	Studies comparing two or more treatments, which are designed, conducted, reported and interpreted to minimize systematic errors (bias) and random errors (resulting from the play of chance) in measuring treatment effects
Framework	A structure, composed of elements, designed (at least in part) to support doing something or learning to do something, such as thinking critically or learning to think critically about claims, comparisons, and choices
Intervention	Any action intended to achieve a goal
Skill	The ability to do something
Thinking critically	Using appropriate criteria (standards for judgment, or principles for evaluation) to make judgements; for example, about the trustworthiness of claims and comparisons, and what to do
Treatment	Any action intended to improve or maintain the health of individuals or communities

## Objective

The objective of our review was to systematically compare the IHC Key Concepts Framework to other frameworks that are relevant to teaching and learning how to think critically about treatment claims, evidence, and choices. We examined similarities and differences between the IHC Key Concepts Framework and other frameworks - particularly in the context of primary and secondary school education - including:

- The purposes and definitions of key terms
- The elements included and domains in which they are grouped
- How the frameworks have been developed and evaluated
- How the frameworks have been used to develop curricula, teaching and learning resources, and assessment tools

## Methods

We conducted a systematic review of frameworks relevant to teaching and learning to think critically about treatment claims, evidence used to support those claims, and choices. The protocol for the review is published on our website ^27^.

### Criteria for considering frameworks for inclusion

We included frameworks that are intended to provide a structure for teaching or learning to think critically about at least one of the following:

- The basis (justification) for claims or arguments about the effects of interventions and the reliability of those justifications
- The extent to which evidence used to support claims about the effects of interventions (comparisons) is fair and reliable
- Choices about what to do in order to achieve a goal

To be included, the sources for each framework had to include:

- a description of the purpose of the framework;
- a list of the framework’s elements; and
- definitions of the key terms used to describe the purpose of the framework, its elements and domains (in which elements are grouped, if there are any).

Frameworks that are modifications of another framework were considered together with the framework that had been modified.

### Search methods for identification of frameworks

We began by considering 41 frameworks reviewed in *Frameworks for Thinking: A Handbook for Teaching and Learning* ^12^ and frameworks with which we were already familiar ^21– 35^. We searched for other relevant frameworks using Google Scholar between October 2018 and June 2019 using the search strategies found in *Extended data File 1*. We supplemented these searches by conducting citation searches and contacting key informants for each category of the frameworks.

### Selection of frameworks

One review author (ADO) initially screened frameworks for possible inclusion. Both review authors then independently assessed full-text articles for each potentially relevant framework using an eligibility form ( *Extended data File 2*). We discussed disagreements and reached a consensus. Frameworks that were assessed for inclusion by both authors and then excluded are listed with the reasons for exclusion in Table 5.

**Table 5.** Excluded frameworks.

Framework	Reason for exclusion	Notes
Bloom taxonomy ^12^	Does not provide a framework for thinking critically about claims, comparisons or choices	This framework is a way of classifying educational goals in terms of complexity. The initial aim was promoting “the exchange of test materials and ideas about testing’ and of ‘stimulating research on examining and on the relations between examining and education” ( 12, p. 49). Bloom’s taxonomy consists of six levels and has a varying amount of detail in the form of sub-categories for each level. The IHC Key Concepts fit into the top level in the original framework - “evaluation”.
Altshuller’s TRIZ Theory of Inventive Problem Solving ^12^	Does not provide a framework for thinking critically about claims, comparisons or choices	“TRIZ is a systematic, creativity and innovation process devised as an aid to practical problem-solving, especially in engineering.” ( 12, p. 122).
De Bono’s lateral and parallel thinking tools ^12^	Does not provide a framework for thinking critically about claims, comparisons or choices	The emphasis of this framework is on problem-solving techniques which promote generative, or productive thinking ( 12, p. 133).
Jewell’s reasoning taxonomy for gifted children ^12^	Does not provide a framework for thinking critically about claims, comparisons or choices	This taxonomy is presented, largely from a philosophical perspective, in response to a perceived need to understand how gifted students think and reason. ( 12, p. 170).
Petty’s six-phase model of the creative process ^12^	Does not provide a framework for thinking critically about claims, comparisons or choices	Consists of six phases: “inspiration; clarification; evaluation; distillation; incubation; and perspiration” ( 12, p. 175).
Bailin’s intellectual resources for critical thinking ^12, 37^	Does not provide a framework for thinking critically about claims, comparisons or choices	Aims at establishing clarity regarding the concept of critical thinking and suggests proposals for an appropriate pedagogy. ( 12, p. 178). Focus is on “intellectual resources” for critical thinking, which includes “knowledge of key critical concepts”, but these are not specified ^37^.
American Philosophical Association (APA) critical thinking consensus ^38^	Does not provide a framework for thinking critically about claims, comparisons or choices	This is a broad framework of skills and dispositions with marginal details relevant to thinking critically about claims, comparisons or choices.
Scientific Discovery as Dual Search (SDDS) model of scientific reasoning ^39^	Does not provide a framework for thinking critically about claims, comparisons or choices	A description of learner behaviour in complex domains. The main ingredients of this model are an elaboration of the “hypothesis space” and “experiment space”, and a representation of learners’ knowledge states during discovery.
Styles of reasoning framework ^15^	Does not provide a framework for thinking critically about claims, comparisons or choices	This is a broad framework that only indirectly addresses judgments about claims and comparisons.
Scaffolding framework for evidence-based arguments ^40, 41^	Does not provide a framework fo thinking critically about claims, comparisons or choices	Provides the basis for a website that supports formulating claims and evidence to support claims but does not provide a framework with support for making judgements about the extent to which evidence used to support claims about the effects of interventions is trustworthy.
Kuhn’s developmental model of critical thinking ^42– 47^	This framework is considered together with related epistemological models	Focuses on how individuals respond to every day, ill-structured problems that lack definitive solutions.
King and Kitchener’s reflective judgment model ^42, 48^	This framework is considered together with related epistemological models	Focuses on the epistemic assumptions that underlie reasoning.
Problem solving ^49^	This framework is considered together with Baron’s model of the good thinker ^36^	Conceptual model of the well-structured problem-solving process.

### Data collection and assessment of included frameworks

For each included framework, we compiled a list of publications that describe the framework, its development and evaluation, and its use as the basis for curricula, learning resources, and assessment tools.

We recorded independently the following information for each framework, using a data collection form ( *Extended data File 3*):

- Its purpose
- Its domains and elements
- Definitions of key terms used to describe its purpose, domains, or elements
- Methods used to develop the framework
- Methods used to evaluate the framework (if any), and findings
- Ways in which the framework has been used as the basis for
  - Curricula
  - Teaching and learning
  - Assessment tools

We compared the data that each of us had collected, discussed disagreements, and reached a consensus.

Based on this information, we assessed independently:

- strengths and weaknesses of how each framework had been developed and evaluated
- strengths and weaknesses of how each framework has been or could be used
- any other strengths or weaknesses

We compared our assessments, discussed disagreements, and reached a consensus.

### Analysis of the data

1. We summarised key characteristics of the included frameworks in tables.
2. Using Venn diagrams, we mapped the extent to which the purposes of the different frameworks overlap with those of the IHC Key Concepts Framework.
3. We compared the concepts, competences and dispositions in each framework with those in the IHC Key Concepts Framework. We considered separately any elements that could not be categorised as concepts, competences or dispositions.
4. We reflected on our assessments of the frameworks and identified implications for how we might improve the IHC Key Concepts Framework, and its usefulness.

We conducted these analyses independently and then compared our analyses, discussed disagreements, and reached consensus.

## Results

**Figure 1.**
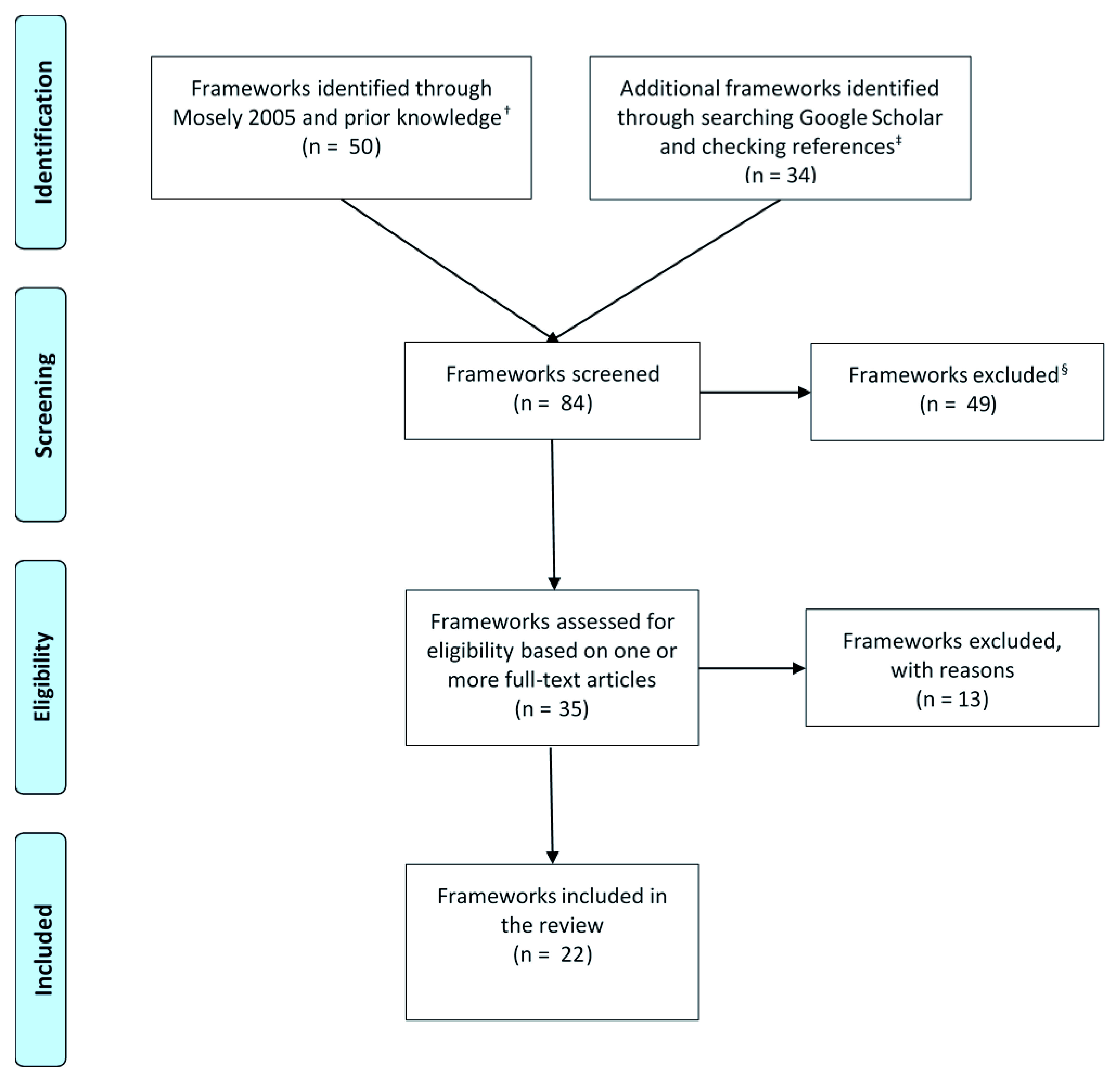
Flow diagram. Frameworks that we grouped together (e.g. health literacy frameworks) are counted as single frameworks. † Frameworks for Thinking: A Handbook for Teaching and Learning (Mosely 2005) has 41 frameworks. ‡ Our primary Google Scholar searches yielded 1588 records. § These frameworks were excluded after being scanned by one of the review authors (ADO).

We screened over 1600 references retrieved using Google Scholar (search strategy: *Extended data File 1*). In addition, we screened the reference lists in the articles that we retrieved. We identified over 80 frameworks and assessed 35 of these for eligibility based on one or more full-text articles ( Figure 1). We excluded 13 of these ( Table 5), so ended up including 22 frameworks ( Table 6).

**Table 6.** Included frameworks.

Framework, who developed it, and when	Purpose	Background
Critical thinking		
**Taxonomy of critical thinking dispositions** **and abilities** Robert Ennis, Emeritus Professor of Philosophy of Education, University of Illinois, USA 1960's ^12, 50– 59^	A set of comprehensive goals for a critical thinking curriculum and its assessment. In deciding what to believe or do, one is helped by having and reflectively employing this set of critical thinking dispositions and abilities.	In 1951 Robert Ennis, then a high school science teacher, tried to infuse critical thinking into his instruction. The trouble was that he did not know what critical thinking was, how to teach it, nor how to test for it. He has worked on these problems throughout his ensuing academic career.
**Model of critical thinking** Richard Paul, a philosopher and founder of the Center for Critical Thinking at Sonoma State University in California and the Foundation for Critical Thinking, USA; and others 1980's ^12, 60– 66^	To help you achieve your goals and ambitions, make better decisions, and understand where others are trying to influence your thinking	“The Center for Critical Thinking and Moral Critique and the Foundation for Critical Thinking — two sister educational non-profit organizations — work closely together to promote educational reform. We seek to promote essential change in education and society through the cultivation of fair-minded critical thinking.”
**List of critical thinking skills** Diane Halpern, Professor of Psychology, Claremont McKenna College, USA 1980's ^12, 67, 68^	Critical thinking skills are those strategies for finding ways to reach a goal.	The list is based on a book published in 1984. The original taxonomy was intended to provide a basis for the national assessment of critical thinking skills in adults in the US. Halpern subsequently revised her taxonomy and presented it, not as a taxonomy, but as a list.
**Model of the good thinker** Jonathan Baron, Department of Psychology, University of Pennsylvania, USA 1980's ^12, 36, 69– 75^	Using a normative theory of the nature of good thinking and of how we tend to think poorly to evaluate our actual thinking, and to know how it must be improved. In this way, we can learn to think more rationally, that is, in a way that helps us achieve our goals.	To arrive at a prescriptive model, we ought to find out where people depart from the normative model. Then we can give practical advice to correct these departures.
Logic and argumentation		
**Logical fallacies** Aristotle, Richard Whately, John Stuart Mill, and others 300's BCE ^35, 68, 76– 84^	A logical fallacy is a flaw in reasoning. If you are aware of these, you will be better prepared to recognize and defend against them.	There are many lists and different ways of classifying logical fallacies, dating back to Aristotle.
**Taxonomy of concepts and critical abilities** **related to the evaluation of verbal arguments** Ronald Allen and a team of educators at the Research and Development Center for Cognitive Learning, University of Wisconsin, USA 1967 ^12, 85, 86^	To identify concepts and clusters of concepts which adequately define what knowledge a student must possess if he is to critically evaluate everyday discourse.	The authors took a "view of argument" derived from Toulmin's presentation of inference as a rule-constituted activity and from the nature of the field of ordinary discourse. It is an analysis of concepts related to the evaluation of ordinary argument, relevant to educators concerned with the development of critical thinking skills.
**Evidence based reasoning framework** Nathaniel Brown, Education Research, Measurement, and Evaluation, Lynch School of Education, Boston College and four colleagues with interests in assessment in science and STEM education, USA 2010 ^87^	To create an analytic tool intended as a foundation for assessing students’ ability to reason from evidence in writing and classroom discussions. This framework is intended to serve many purposes in the elementary, middle, and high school science classroom, including: (a) supporting students’ and teachers’ understanding of the process of scientific reasoning; (b) modelling exemplary scientific reasoning; (c) diagnosing problems and identifying pitfalls affecting student reasoning as it develops; and (d) assessing scientific reasoning in the classroom both formatively and summatively.	The authors chose not to apply Toulmin’s framework directly to scientific arguments. Instead, they simplified Toulmin’s framework and then adapted it to incorporate what is currently known about the process of scientific inquiry. They synthesized Toulmin's and Duschl's frameworks to create a framework of scientific reasoning as a distinct mode of thought and discourse with roots in both general argumentation and scientific inquiry.
Cognition		
**Cognitive biases** Amos Tversky, cognitive and mathematical psychologist and Daniel Kahneman, psychologist and economist, Israel and USA; and others 1970's ^36, 88– 96^	To study and document biases of intuitive thinking in various tasks or beliefs concerning uncertain events. People rely on a limited number of heuristic principles which reduce the complex tasks of assessing probabilities and predicting values to simpler judgmental operations. In general, these heuristics are quite useful, but sometimes they lead to severe and systematic errors.	Tversky and Kahneman are recognised as the founders of cognitive bias theory and their 1974 *Science* paper was the first codification of the area. They based their classification on their own theory of general judgemental heuristics. The basis for different classifications varies, but they all are based, at least in part, on research evidence of the existence of the included biases.
**Framework for understanding people's** **theories about their own cognition** John Flavell, developmental psychologist specializing in children's cognitive development, USA; Gregory Schraw and David Moshman, Department of Educational Psychology, University of Nebraska, USA; and others 1970's ^97– 107^	To consider how individuals consolidate different kinds of metacognitive knowledge and regulatory skills into systematized cognitive frameworks, the origin and development of those, and implications for educational research and practice.	Schraw and Moshman ^103^ reviewed standard accounts of metacognition and how metacognitive knowledge and regulation affect cognitive performance. Metacognition, which has been defined in different ways, refers to both knowledge of cognition (an awareness of variables that influence thinking) and regulation of cognition (the ability to regulate one's learning). it is sometimes defined as thinking about thinking.
**Epistemological models** Jean Piaget, development psychologist, Switzerland; William Perry Jr., educational psychologist, Harvard, USA; and others 1950's ^42– 48, 108– 113^	To describe changes in assumptions about sources and certainty of knowledge (the development of epistemic assumptions) and how decisions are justified in light of those assumptions (how epistemic assumptions affect the way individuals understand and solve problems).	Epistemology is an area of philosophy concerned with the nature and justification of human knowledge. A growing area of interest for psychologists and educators is that of personal epistemological development and epistemological beliefs: how individuals come to know, the theories and beliefs they hold about knowing, and the manner in which such epistemological premises are a part of and an influence on the cognitive processes of thinking and reasoning.
**AIR model of epistemic cognition** Ravit Duncan, Clark Chinn, Luke Buckland, Graduate School of Education, Rutgers University, USA; Sarit Barzilai, Faculty of Education, University of Haifa, Israel; Ronald Rinehart, Department of educational Psychology and Foundations, University of Northern Iowa, USA 2014 ^114– 117^	To help account for how people evaluate information, including inaccurate information and the role that cognitions play in people’s evaluation of inaccurate (as well as accurate) information.	Educational and developmental psychologists have investigated human cognitions about epistemic matters. These are cognitions about a network of interrelated topics including knowledge, its sources and justification, belief, evidence, truth, understanding, explanation, and many others. Different researchers have used different terms for these cognitions, including personal epistemology, epistemological beliefs, epistemic beliefs, epistemic positions, epistemic cognition, epistemological reflection, and reflective judgment.
Scientific thinking		
**PISA framework for scientific literacy** Organisation for Economic Co-operation and Development (OECD). The Programme for International Student Assessment (PISA) is a collaborative effort among the OECD member governments to provide a new kind of assessment of student achievement on a recurring basis. 1997 ^118– 124^	The main benefit of constructing and validating the framework is improved measurement. Other potential benefits include: a common language, an analysis of the kinds of knowledge and skills associated with successful performance, and identifying and understanding particular variables that underlie successful performance.	PISA is designed to collect information through three-yearly cycles and presents data on the reading, mathematical and scientific literacy of 15-year-old students, schools and countries. It provides insights into the factors that influence the development of skills and attitudes at home and at school, and examines how these factors interact and what the implications are for policy development.
**Framework for K-12 science education** National Research Council (NRC) Committee on a Conceptual Framework for New K-12 Science Education Standards, USA. The committee included professionals in the natural sciences, mathematics, engineering, cognitive and developmental psychology, the learning sciences, education policy and implementation, research on learning science in the classroom, and the practice of teaching science. 2010 ^125– 132^	To articulate a broad set of expectations for students in science. The overarching goal is to ensure that by the end of 12th grade, all students have some appreciation of the beauty and wonder of science; possess sufficient knowledge of science and engineering to engage in public discussions on related issues; are careful consumers of scientific and technological information related to their everyday lives; are able to continue to learn about science outside school; and have the skills to enter careers of their choice, including (but not limited to) careers in science, engineering, and technology.	The framework was the first part of a two-stage process to produce a next generation set of science standards for voluntary adoption by states in the USA.
**Systems thinking** Ideas about holistic thinking and change processes can be traced back to the ancient Greeks. The start of modern systems thinking is attributed the articulation of systems ideas by Ludwig von Bertalanffy, an Austrian biologist who started lecturing and writing in the 1930's on what he called “general system theory”; and to Aleksandr Bogdanov, a Russian revolutionary, philosopher and scientist. 1910's ^133– 142^	To understand and interpret complex systems in order to navigate information, make decisions, and solve problems.	Systems theory is the transdisciplinary study of the abstract organisation of phenomena, independent of their substance, type, or spatial and temporal scale. Systems can be used to represent the complex organisation of virtually any collection of real-world entities into an ordered form that we can better understand. There are several conceptualizations of systems thinking in education.
**Model for scientific thinking** Gregory Feist, Department of Psychology, College of William & Mary, USA; Carlo Magno, Counselling and Educational Psychology, De La Salle University, Philippines 1990's ^143, 144^	To investigate the relationship of the constructs scientific thinking, self- regulation in research, and creativity in a measurement model.	Feist investigated whether personality traits consistently distinguish artists from non- artists and scientists from non-scientists. Magno ^144^, building on Feist's work ^143^, investigated the relationship between scientific thinking, self-regulation, and creativity.
Evidence-based health care		
**Health literacy frameworks** The term ‘health literacy’ was first coined in 1974 by Scott Simonds, Professor of Health Education, University of Michigan, School of Public Health, USA. Several frameworks have been developed since then. 1970's ^21– 23, 145– 149^	To develop health literacy enhancing interventions and to develop and validate of measurement tools.	Simonds wrote in 1974 that: “Minimum standards for 'health literacy' should be established for all grade levels K through 12. Those school districts that fall below standard should be provided with federal aid to develop programs with teachers qualified to teach health education" ^150, 151^. Since then, it has been estimated that approximately 80 million Americans have limited health literacy, and multiple studies have found that low health literacy is associated with poorer health outcomes and poorer use of health care services ^152^.
**Evidence-based practice (EBP) core** **competencies** International EBP leaders led by team at Bond University, Australia 2018 ^26, 153^	To develop a consensus-based set of core EBP competencies that EBP teaching and learning programs should cover	The term evidence-based medicine was first developed in the field of medicine in the early 1990s, but as its use expanded to include other health disciplines, it became known as EBP. EBP provides a framework for the integration of research evidence and patients’ values and preferences into the delivery of health care. Although many teaching strategies have been used and evaluated, a lack of EBP knowledge and skills is still one of the most commonly reported barriers to practicing EBP. One of the potential explanations is the inconsistency in the quality and content of the EBP teaching programs.
**GRADE (and related frameworks)** The GRADE Working Group, which includes methodologists, health researchers, systematic review authors, guideline developers 2000 ^25, 30, 154– 160^	Grading of Recommendations Assessment, Development, and Evaluation (GRADE) offers a transparent and structured process for developing and presenting summaries of evidence, including its quality, for systematic reviews and recommendations in health care. The purpose of Evidence to Decision (EtD) frameworks is to help people use evidence in a structured and transparent way to inform decisions in the context of clinical recommendations, coverage decisions, and health system or public health recommendations and decisions.	Since the 1970s a growing number of organisations have employed various systems to grade the quality (level) of evidence and the strength of recommendations. Different organisations have used different systems, resulting in confusion and impeding effective communication. The GRADE Working Group began as an informal collaboration of people with an interest in tackling the shortcomings of prior grading systems.
**Bradford-Hill criteria** Austin Bradford Hill, Professor Emeritus of Medical Statistics, University of London, UK 1965 ^28, 161– 165^	To address: "What aspects of an association between two variables should we especially consider before deciding that the most likely interpretation of it is causation?"	This framework was developed to identify the causes of diseases and particularly to determine the role of smoking in lung cancer, but its use has been extended to public health decision making, a domain where questions about causal effects relate to the consequences of interventions that have often been motivated by the identification of causal factors. It has proven useful and has driven decision making in public health for decades.
**Critical appraisal** International teachers of evidence-based health care and research methodologists 1981 ^29, 166– 179^	To teach critical appraisal of health research. However, some critical appraisal tools are intended primarily for critically appraising research in the context of systematic reviews and some are intended primarily for reporting standards. There is an overlap among these tools and clear distinctions are sometimes not made among tools with different purposes.	“The strategies we shall suggest assume that clinical readers are already behind in their reading and that they will never have more time to read than they do now. For this reason, and because the guides that follow call for closer attention to "Materials and methods" and other matters that often appear in small type, many of the guides recommend tossing an article aside as not worth reading, usually on the basis of quite preliminary evidence. It is only through the early rejection of most articles that busy clinicians can focus on the few that are both valid and applicable in their own practices.” ^170^
**Cochrane risk of bias tool (and related** **frameworks)** International health research methodologists 1980's ^29, 31, 33, 180– 184^	To assess the risk of bias in randomised and non-randomised studies (sometimes referred to as quality or internal validity). Assessments of risk of bias are intended to help interpret findings and explain heterogeneity in systematic reviews; in addition, reviews use risk-of-bias assessments of individual studies in grading the certainty of the evidence. Reviews may exclude studies assessed as high risk of bias.	"The concern about study quality first arose in the early 1980s with the publication of a landmark paper by Tom Chalmers and colleagues and another extensive work by Hemminki, who evaluated the quality of trials done in 1965 through 1975 that were used to support the licensing of drugs in Finland and Sweden ^185^.
**Catalogue of biases** Centre for Evidence Based Medicine, Oxford University, UK 2017 ^34^	To obtain the least biased information, researchers must acknowledge the potential presence of biases and take steps to avoid and minimise their effects. Equally, in assessing the results of studies, we must be aware of the different types of biases, their potential impact and how this affects interpretation and use of evidence in health care decision making. To better understand the persistent presence, diversity, and impact of biases, we are compiling a Catalogue of Biases, stemming from original work by David Sackett. The entries are a work in progress and describe a wide range of biases – outlining their potential impact in research studies.	David Sackett, in his 1979 paper “Bias in Analytic Research” ^186^, reported the first draft of a ‘catalog of biases which may distort the design, execution, analysis, and interpretation of research.’ Sackett catalogued 35 biases that arise in the context of clinical trials and listed 56 biases potentially affecting case-control and cohort studies. He proposed the continued development of an annotated catalogue of bias as a priority for research. He suggested that each citation should include a useful definition, a referenced example illustrating the magnitude and direction of its effects, and a description of the appropriate preventive measures if any.

We included four frameworks on critical thinking, three on logic and argumentation, four on cognition, four on scientific thinking, and seven on evidence-based healthcare. We grouped several frameworks together for five types of frameworks - logical fallacies, cognitive biases, epistemological models, systems thinking, and health literacy. We also considered related frameworks together with the *Grading of Recommendations Assessment, Development, and Evaluation (GRADE) framework* and the *Cochrane Risk of Bias Tool*. The purpose and background of each of the included frameworks are shown in Table 6, and definitions of the key term for each framework are shown in Table 7.

**Table 7.** Definitions of the core term for each included framework.

Frameworks	Definitions
Critical thinking	
Taxonomy of critical thinking dispositions and abilities	Critical thinking is “reasonable reflective thinking focused on deciding what to believe or do. http://criticalthinking.net/index.php/longdefinition/
Model of critical thinking	Critical thinking is the intellectually disciplined process of actively and skilfully conceptualizing, applying, analysing, synthesizing, and/or evaluating information gathered from, or generated by, observation, experience, reflection, reasoning, or communication, as a guide to belief and action. http://www.criticalthinking.org/pages/defining-critical-thinking/766
List of critical thinking skills	Critical thinking is the use of those cognitive skills or strategies that increase the probability of a desirable outcome. It is purposeful, reasonable, and goal directed. Also known as directed thinking ^68^.
Model of the good thinker	The definition of rationality as “the kind of thinking that helps us achieve our goals. A good decision is one that makes effective use of the information available to the decision maker at the time the decision is made. A good outcome is one that the decision maker likes. The whole point of good thinking is to increase the probability of good outcomes (and true conclusions), but many other factors affect outcomes aside from good thinking. Good decision making involves sufficient search for possibilities, evidence, and goals, and fairness in the search for evidence and in inference ^36^.
Logic and argumentation	
Logical fallacies	Fallacy is the use of invalid or otherwise faulty reasoning in the construction of an argument. https://en.wikipedia.org/wiki/Fallacy
Taxonomy of concepts and critical abilities related to the evaluation of verbal arguments	The evaluation of verbal arguments is the process of applying higher-order concepts (i.e., rules or principles concerning the nature, structure, and tests of argument) to arguments occurring in ordinary verbal discourse in order to assess their acceptability. Such an evaluation requires that one understand numerous concepts and employ diverse critical abilities ^85^.
Evidence based reasoning framework	To participate in arguments about scientific ideas, students must learn how to evaluate and use evidence. That is, apart from what they may already know about the substance of an assertion, students who are scientifically literate should be able to make judgments based on the evidence supporting or refuting that assertion ^87^.
Cognition	
Cognitive biases	Cognitive biases are systematic patterns of deviation from norm or rationality in judgment. https://en.wikipedia.org/wiki/List_of_cognitive_biases
Framework for understanding people's theories about their own cognition	Metacognitive theories are theories that integrate one's knowledge about cognition and regulation of cognition. By "theory" we mean a relatively systematic structure of knowledge that can be used to explain and predict a broad range of empirical phenomena. By a "metacognitive theory" we mean a relatively systematic structure of knowledge that can be used to explain and predict a broad range of cognitive and metacognitive phenomena ^103^.
Epistemological models	Definitions of critical thinking are numerous and wide-ranging. However, one non-controversial claim we can make about critical thinking is that it entails awareness of one’s own thinking and reflection on the thinking of self and others as an object of cognition. Metacognition, a construct that is assuming an increasingly central place in cognitive development research, is defined in similar terms as awareness and management of one’s own thought, or “thinking about thinking.” Metacognition originates early in life, when children first become aware of their own and others’ minds. But like many other intellectual skills, metacognitive skills typically do not develop to the level we would like ^47^.
AIR model of epistemic cognition	Epistemic cognition refers to the complex of cognitions that are related to the achievement of epistemic ends; notable epistemic ends include knowledge, understanding, useful models, explanations, and the like ^116^.
Scientific thinking	
PISA framework for scientific literacy	Scientific literacy is an individual’s scientific knowledge and use of that knowledge to identify questions, to acquire new knowledge, to explain scientific phenomena, and to draw evidence-based conclusions about science-related issues, understanding of the characteristic features of science as a form of human knowledge and enquiry, awareness of how science and technology shape our material, intellectual, and cultural environments, and willingness to engage in science-related issues, and with the ideas of science, as a reflective citizen ^122^.
Framework for K-12 science education	Science, engineering, and the technologies they influence permeate every aspect of modern life. Indeed, some knowledge of science and engineering is required to engage with the major public policy issues of today as well as to make informed everyday decisions, such as selecting among alternative medical treatments or determining how to invest public funds for water supply options. In addition, understanding science and the extraordinary insights it has produced can be meaningful and relevant on a personal level, opening new worlds to explore and offering lifelong opportunities for enriching people’s lives. In these contexts, learning science is important for everyone, even those who eventually choose careers in fields other than science or engineering. By framework we mean a broad description of the content and sequence of learning expected of all students by the completion of high school—but not at the level of detail of grade-by-grade standards or, at the high school level, course descriptions and standards. Instead, as this document lays out, the framework is intended as a guide to standards developers as well as for curriculum designers, assessment developers, state and district science administrators, professionals responsible for science teacher education, and science educators working in informal settings ^32^.
Systems thinking	System thinking is the ability to understand and interpret complex systems. Our conceptualisation of systems thinking is based on Riess and Mischo’s definition: “as the ability to recognise, describe, model (e.g. to structure, to organise) and to explain complex aspects of reality as systems”. According to this definition, Riess and Mischo stressed essential aspects of systems thinking, which include the ability to identify important elements of systems and the varied interdependency between these elements, the ability to recognise dimensions of time dynamics, the ability to construct an internal model of reality and the ability to give explanations, to make prognoses and to develop means and strategies of action based on that model ^141^.
Model for scientific thinking	Scientific thinking is composed of a set of characteristics that includes practical inclination, analytical interest, intellectual independence, and assertiveness ^144^. Broadly defined, scientific thinking includes the skills involved in inquiry, experimentation, evidence evaluation, and inference that are done in the service of conceptual change or scientific understanding. Scientific thinking is defined as the application of the methods or principles of scientific inquiry to reasoning or problem-solving situations, and involves the skills implicated in generating, testing and revising theories, and in the case of fully developed skills, to reflect on the process of knowledge acquisition and change. Participants engage in some or all the components of scientific inquiry, such as designing experiments, evaluating evidence and making inferences ^16^.
Evidence-based health care	
Health literacy frameworks	There are various definitions of health literacy. A “new ‘all inclusive’ comprehensive definition capturing the essence of the 17 definitions identified in the literature” is: Health literacy is linked to literacy and entails people’s knowledge, motivation and competences to access, understand, appraise, and apply health information to make judgments and take decisions in everyday life concerning healthcare, disease prevention and health promotion to maintain or improve quality of life during the life course ^22^.
EBP core competencies	Evidence-Based Practice (EBP) is the integration of the best research evidence with clinical expertise and patient’s unique values and circumstances. Core competencies are defined as the essential minimal set of a combination of attributes, such as applied knowledge, skills, and attitudes, that enable an individual to perform a set of tasks to an appropriate standard efficiently and effectively ^26^.
GRADE and related frameworks	Quality of evidence (also referred to as certainty of the evidence or certainty of the anticipated effect) is the extent to which one can be confident that an estimate of effect is correct. Strength of the recommendation is the degree of confidence that the desirable effects of adherence to a recommendation outweigh the undesirable effects. https://gdt.gradepro.org/app/handbook/handbook.html#h.svwngs6pm0f2
Bradford Hill criteria	An association (or correlation) in statistics is a relationship between two variables in a study, e.g. between having received a particular treatment and having experienced a particular outcome. Causation (a causal association) is an association between two variables where a change in one makes a change in the other one happen. http://getitglossary.org/
Critical appraisal	"Critical appraisal is the systematic evaluation of clinical research papers in order to establish: 1. Does this study address a clearly focused question? 2. Did the study use valid methods to address this question? 3. Are the valid results of this study important? 4. Are these valid, important results applicable to my patient or population?" https://www.cebm.net/2014/06/critical-appraisal/
Risk of bias	Bias is the result of “flaws in design, conduct, analyses, and reporting, leading to underestimation or overestimation of the true intervention effect”. “It is usually impossible to know the extent to which biases have affected the results of a particular trial” ^31^.
Catalogue of biases	Biases (systematic errors) distort effect estimates away from actual effects. Biases are caused by inadequacies in the design, conduct, analysis, reporting, or interpretation of treatment comparisons. Because it is generally not possible to know the degree to which an effect estimate is biased, judgements must be made about the risk of bias using criteria that assess factors that are known, or thought to be associated with bias, such as unconcealed allocation of participants to treatments. In everyday language, bias has other meanings, for example ’prejudice’. http://getitglossary.org/term/bias

### Comparison of the included frameworks to the IHC Key Concepts Framework

We summarise our comparison of the included frameworks to the IHC Key Concepts Framework in Table 8. Two frameworks had a similar purpose: Ennis’ *taxonomy of critical thinking dispositions and abilities* ^12, 50– 59^ and Baron’s *model of the good thinker* ^12, 36, 69– 75^. Ennis’ goal is for students to learn to think critically about what to believe or do. Baron’s goal is for students to learn to think more rationally, that is, in a way that helps them to achieve their goals. Both those goals are broader than that of the IHC Key Concepts Framework, which is to enable people to make informed decisions about which claims to believe about the effects of things they can do (interventions) for their health, the health of others or for other reasons, and about what to do to achieve their goals ^4^. The purposes of the two other critical thinking frameworks that we included (the *Model of critical thinking* and *List of critical thinking skills*) were also somewhat like the purpose of the IHC Key Concepts Framework.

**Table 8.** Comparison of included frameworks to the IHC framework.

Framework	Purpose*	Scope	Concepts ^†^	Competences ^†^	Dispositions ^†^
Critical thinking					
Taxonomy of critical thinking dispositions and abilities		Broader	Yes	Yes	Yes
Model of critical thinking		Broader	Yes	Yes	Yes
List of critical thinking skills		Broader	Yes	Yes	Yes
Model of the good thinker		Broader	No	Yes	Yes
Logic and argumentation					
Logical fallacies		Overlapping	Yes	No	No
Taxonomy of concepts and critical abilities related to the evaluation of verbal arguments		Overlapping	Yes	Yes	No
Evidence based reasoning framework		Overlapping	Yes	No	No
Cognition					
Cognitive biases		Overlapping	Yes	No	No
Framework for understanding people's theories about their own cognition		Overlapping	No	Yes	No
Epistemological models		Overlapping	No	No	Yes
AIR model of epistemic cognition		Overlapping	Yes	Yes	Yes
Scientific thinking					
PISA framework for scientific literacy		Overlapping	Yes	Yes	Yes
Framework for K-12 science education		Overlapping	Yes	Yes	No
Systems thinking		Narrower	Yes	Yes	No
Model for scientific thinking		Non-overlapping	No	No	Yes
Evidence-based health care					
Health literacy frameworks		Broader	No	Yes	No
Evidence-based practice (EBP) core competencies		Broader	No	Yes	No
GRADE and related frameworks		Overlapping	Yes	No	No
Bradford-Hill criteria		Overlapping	Yes	No	No
Critical appraisal		Overlapping	Yes	Yes	No
Risk of bias		Narrower	Yes	No	No
Catalogue of biases		Overlapping	Yes	No	No

**Table:** 

* Similarity to the IHC framework:	**Similar**	**Some similarity**	**Little similarity**	**Not similar**

**Table:** 

Overlap with the IHC framework:	**Some overlap**	**Little overlap**	**No overlap**

† Yes = included in the framework; No = not included in the framework

**Figure 2.**
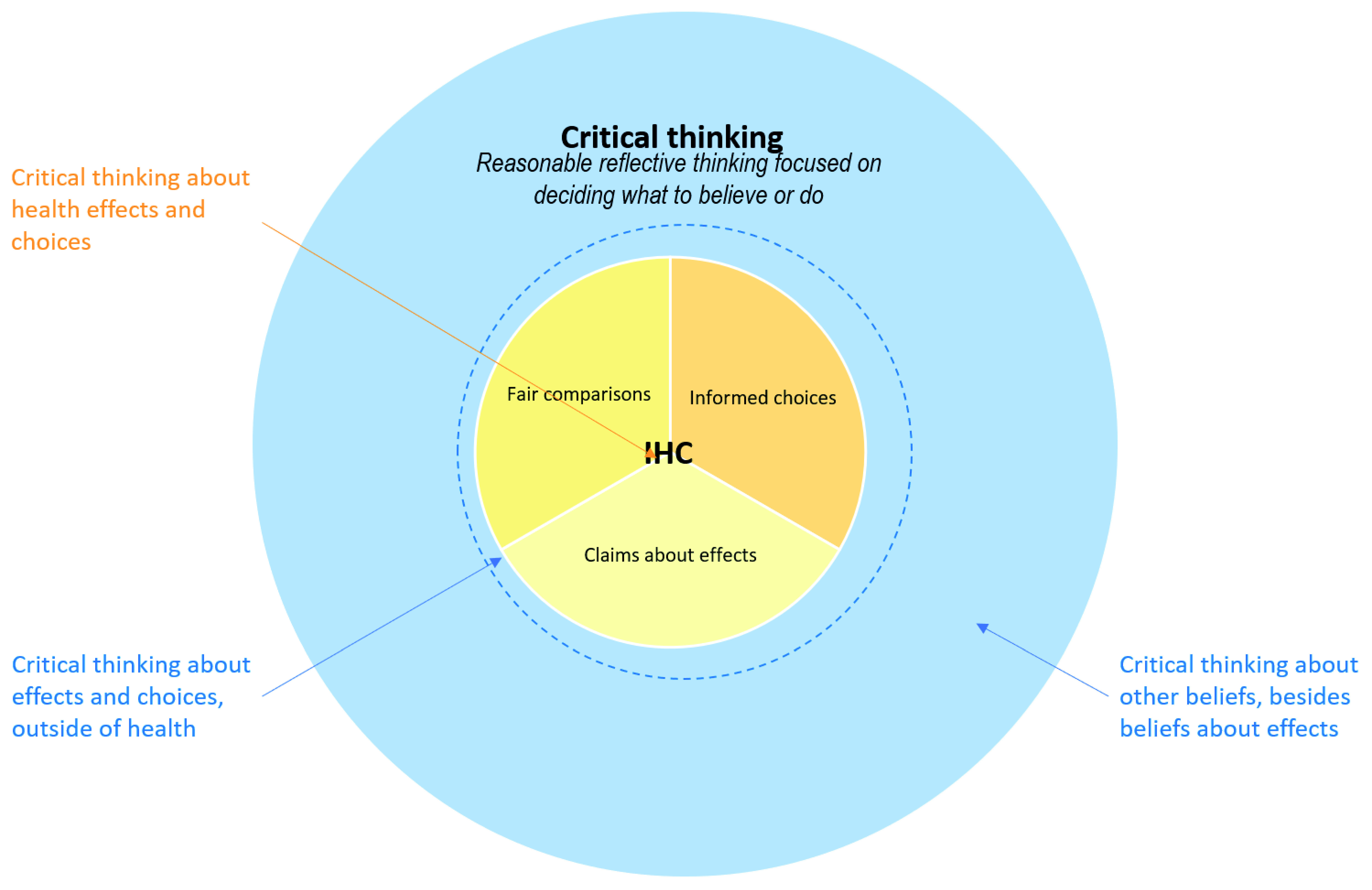
Venn diagram showing the relationship between critical thinking and the IHC framework.

Figure 2 illustrates how we view the relationship between critical thinking and the IHC Key Concepts Framework. Although the IHC framework focuses specifically on critical thinking about health effects and choices, the same Key Concepts can be applied to many other types of interventions (actions) and decisions ^7^. Because achieving our goals depends on what we do (actions), deciding what to believe about the possible effects of our actions and what to do is at the centre of critical thinking. However, critical thinking also applies to many other types of beliefs, such as beliefs about religion, history, or art.

The goal of the IHC Key Concepts Framework is “To enable people to make good decisions about which claims to believe about the effects of things they can do for their health, the health of others or for other reasons, and about what to do to achieve their goals” ^4^. Our formulation of that goal was influenced by how Ennis and Baron formulated their goals. We have adapted Baron’s definition of a “good decision” ^36^ to explain what this means: a good decision is one that makes effective use of the information available to the decision maker at the time the decision is made. A good outcome is one that the decision maker likes. The aim of thinking critically about treatments is to increase the probability of good outcomes (and true conclusions), but many other factors affect outcomes aside from critical thinking.

**Figure 3.**
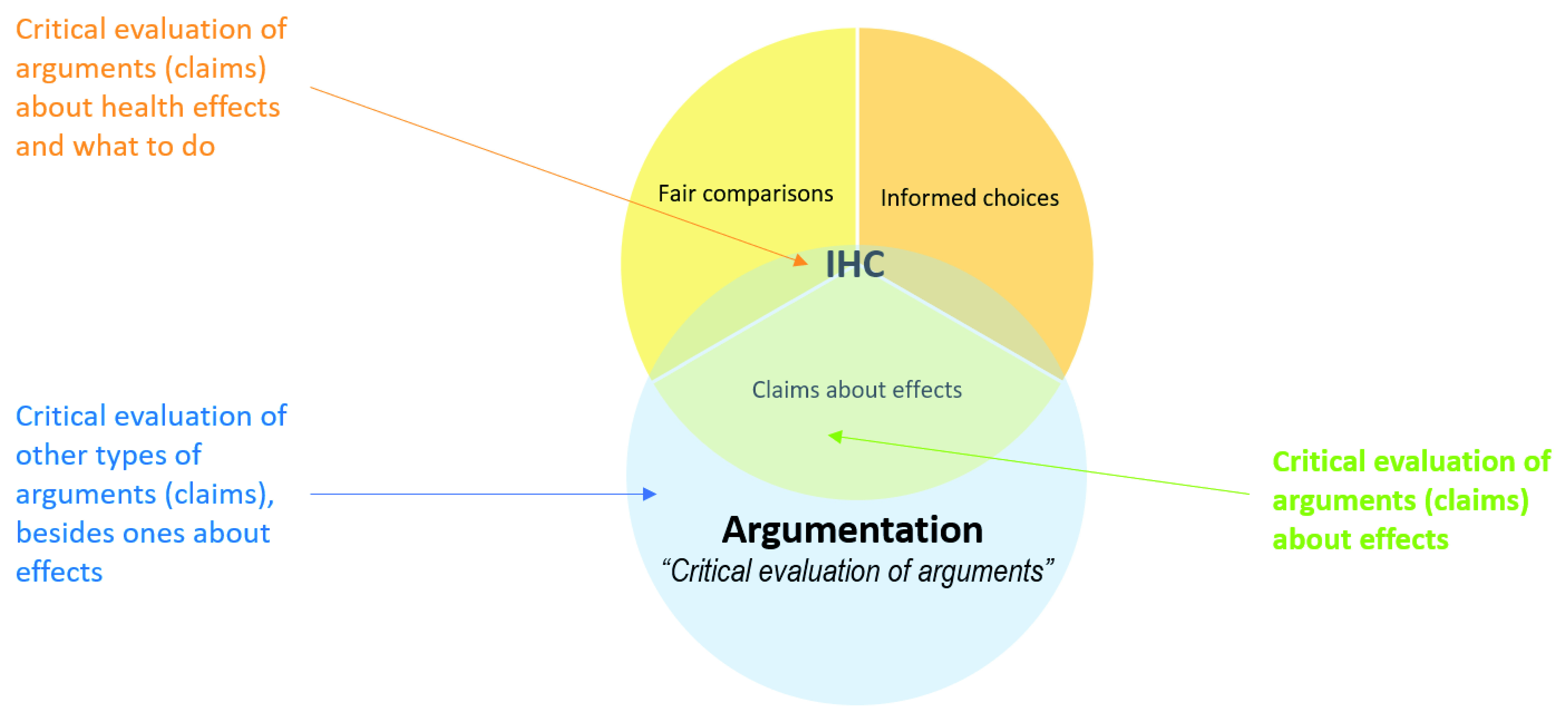
Venn diagram showing the relationship between argumentation and the IHC framework.

The purpose of one of the logic and argumentation frameworks that we included had a somewhat similar purpose to that of the IHC Key Concepts Framework. The *evidence-based reasoning framework* ^87^ was developed as an analytic tool intended as a foundation for assessing students’ ability to reason from evidence in writing and classroom discussions. The relationship between argumentation – critical evaluation of arguments – and the IHC Key Concepts Framework is illustrated in Figure 3. The purposes of four of the evidence-based health care frameworks were also somewhat similar to the purpose of the IHC Key Concepts Framework: health literacy ^21– 23, 145– 149^, the *Evidence-based practice (EBP) core competencies* ^26^, *GRADE* ^25, 30, 154– 160^, and critical appraisal tools ^29, 166– 179^.

**Figure 4.**
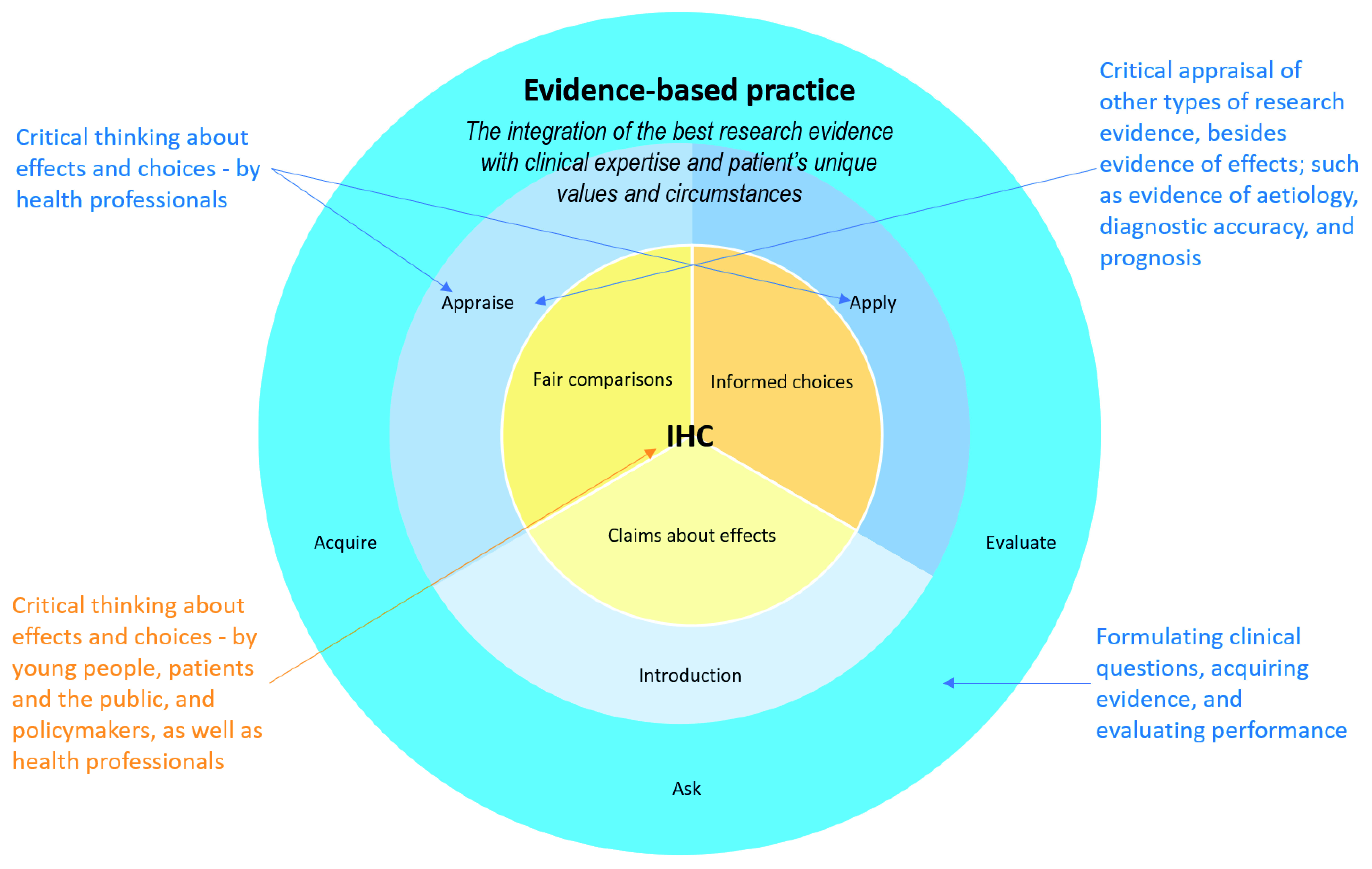
Venn diagram showing the relationship between evidence-based practice and the IHC framework.

Figure 4 illustrates the relationship between the evidence-based practice framework and the IHC Key Concepts Framework. Evidence-based practice is a framework for health professionals, whereas the IHC Key Concepts Framework is for young people, patients and the public, and policymakers, as well as health professionals. Evidence-based practice is a broader framework, which includes critical appraisal of other types of evidence besides evidence of effects. It also includes formulating clinical questions, acquiring evidence, and evaluating performance, which are largely outside of the scope of the IHC Key Concepts Framework. The aim of evidence-based practice is to improve health outcomes, and that depends on what health professionals, patients and the public do. Thus, the IHC Key Concepts Framework – critical thinking about effects and choices – is at the centre of evidence-based practice, in much the same way as it is at the centre of critical thinking.

**Figure 5.**
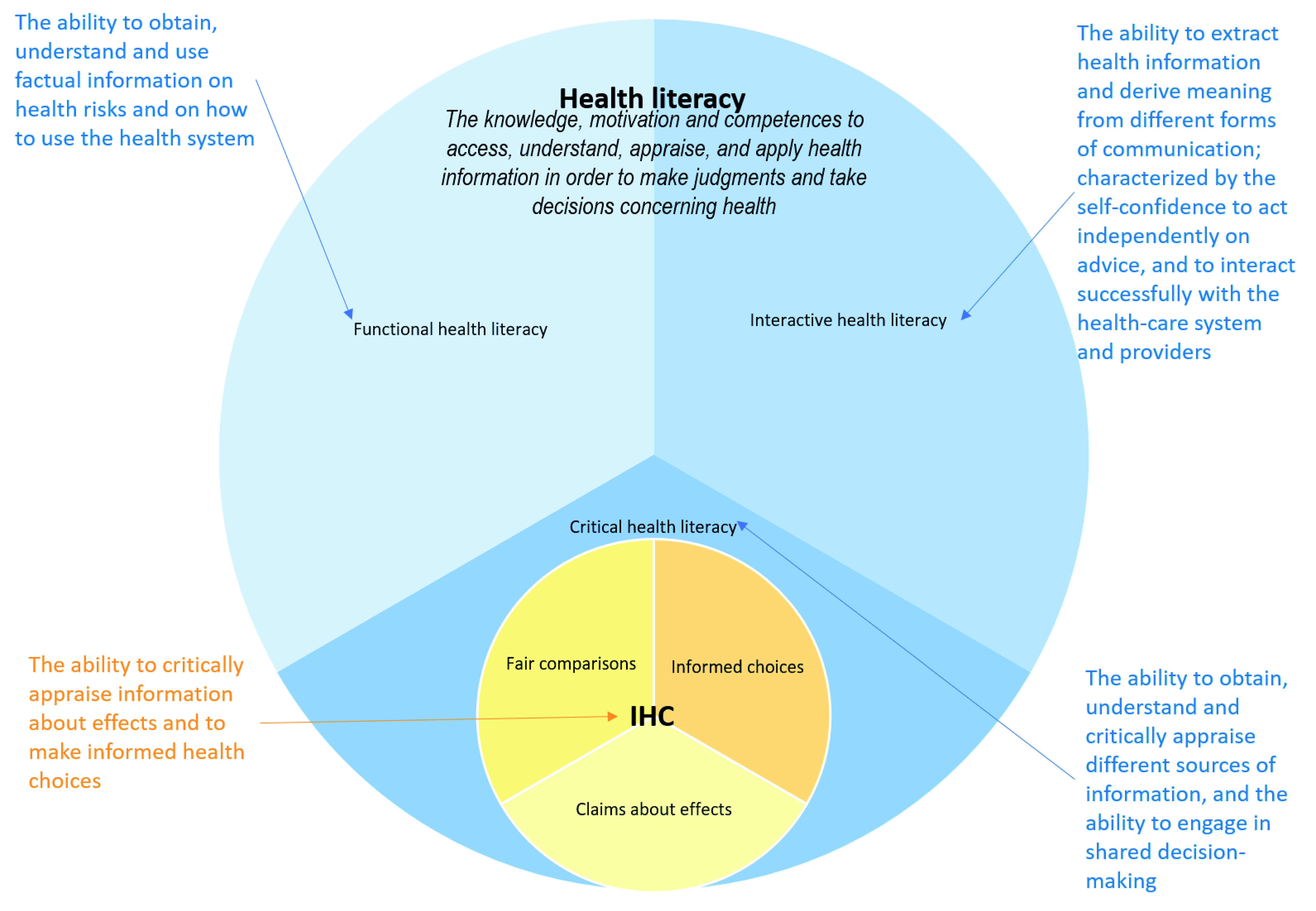
Venn diagram showing the relationship between health literacy and the IHC framework.

Health literacy also has a broader focus than the IHC Key Concepts Framework. This is most clearly illustrated by Nutbeam’s framework ^145, 148^, which divides health literacy into functional, interactive, and critical health literacy. The IHC Key Concepts Framework is most closely related to critical health literacy, as illustrated in Figure 5.

**Figure 6.**
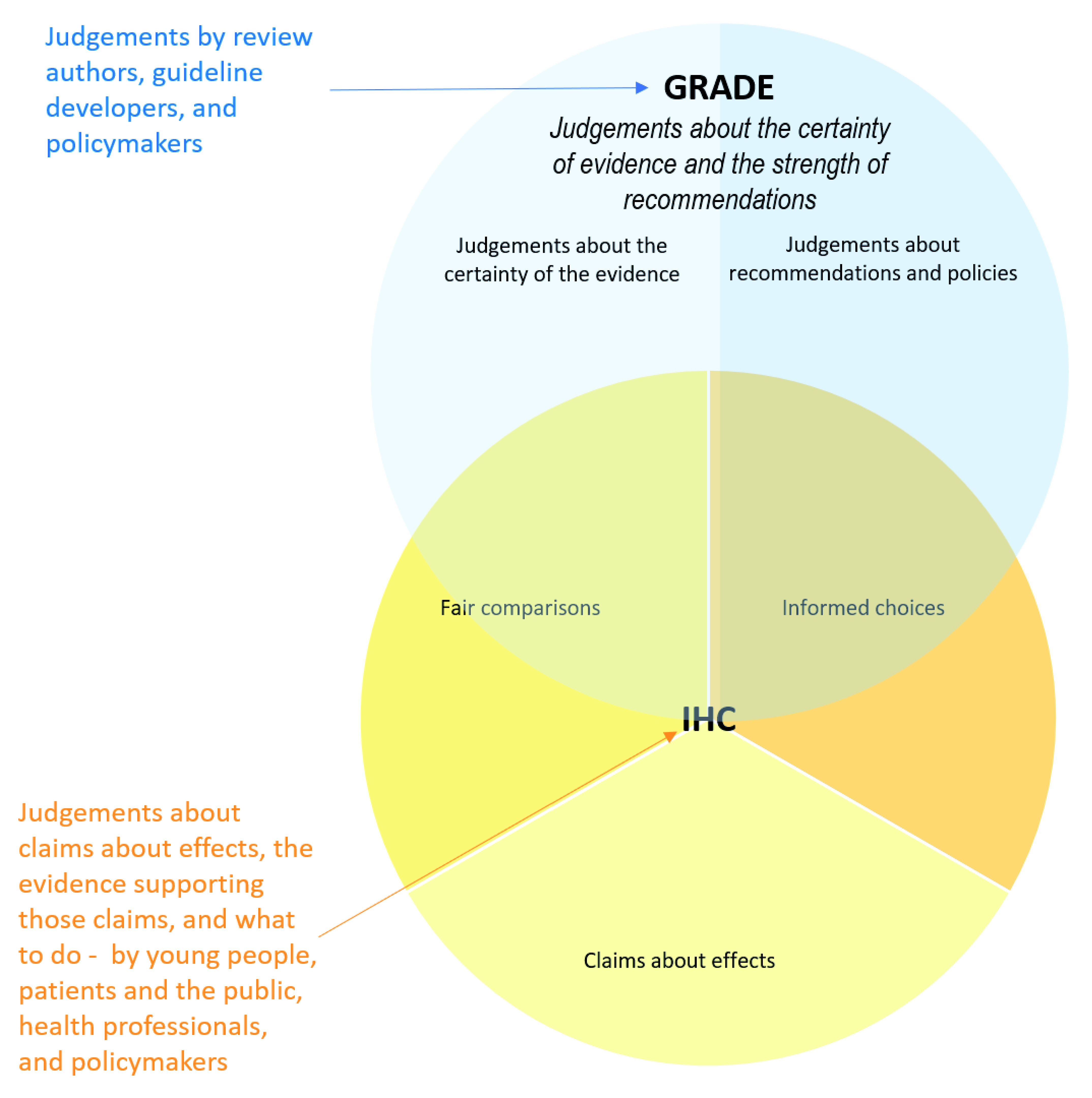
Venn diagram showing the relationship between GRADE and the IHC framework.

The *GRADE framework* overlaps substantially with the IHC Framework with respect to critical thinking about evidence of intervention effects and decisions about what to do, as illustrated in Figure 6. However, the *GRADE framework* is designed primarily for judgements by authors of systematic reviews, guideline developers, and policymakers.

**Figure 7.**
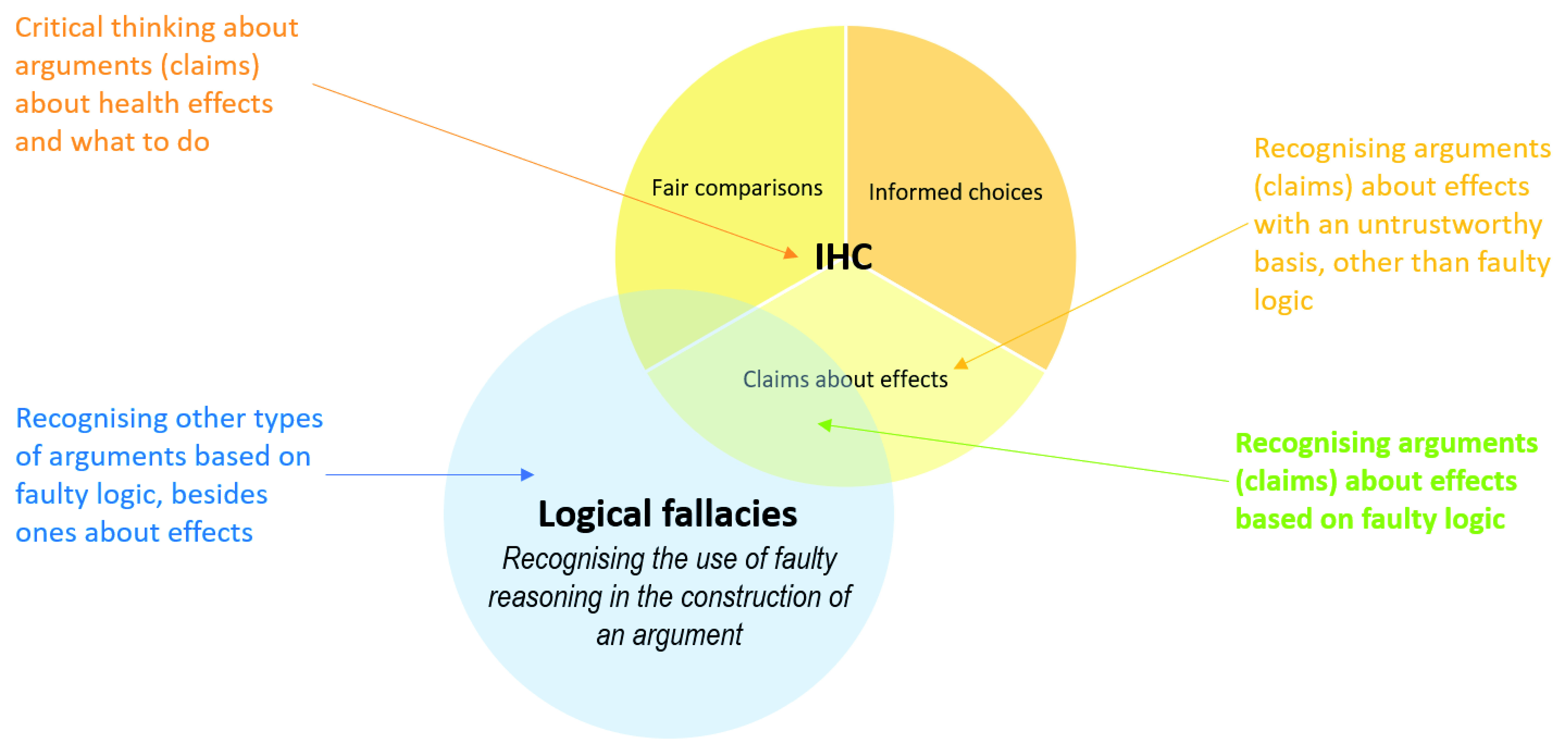
Venn diagram showing the relationship between logical fallacies frameworks and the IHC framework.

**Figure 8.**
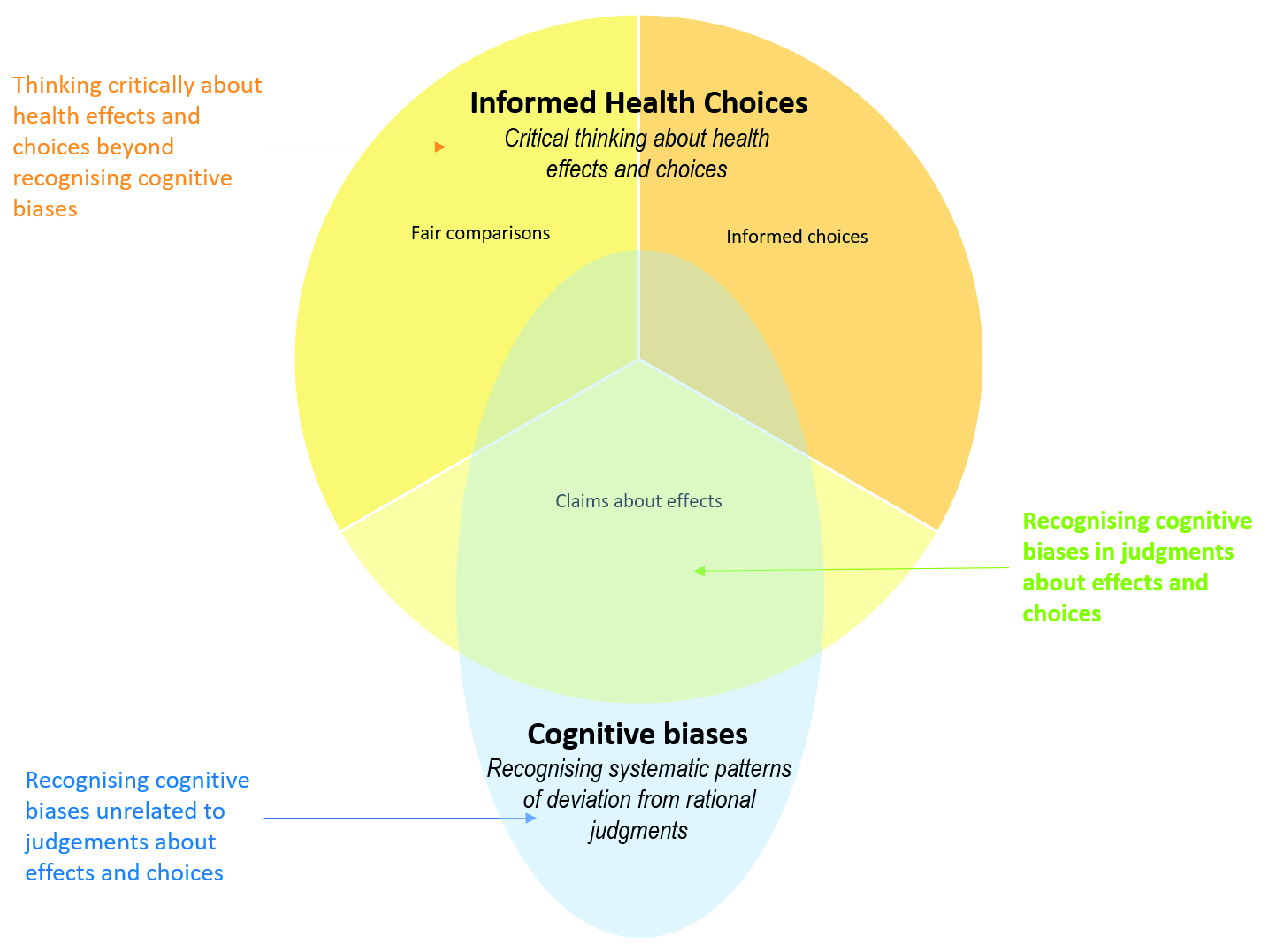
Venn diagram showing the relationship between cognitive biases frameworks and the IHC framework.

Logical fallacies ^35, 68, 76– 84^ and cognitive biases ^36, 88– 96^ are both highly relevant to the IHC Key Concepts Framework. However, there is little similarity between the purposes of either of those types of frameworks and the purpose of the IHC Key Concepts Framework ( Figure 7 and Figure 8). Recognising the use of faulty reasoning in the construction of an argument overlaps with recognising faulty logic underlying claims about effects. However, most logical fallacies are not directly relevant to this. Similarly, recognising systematic patterns of deviation from rational judgements (cognitive biases) overlaps with judgements about effects and choices, but most cognitive biases are not directly relevant. In addition, most of the IHC Key Concepts are not logical fallacies or cognitive biases.

There was at most some overlap between the concepts, competences, and dispositions in the included frameworks and those in the IHC Key Concepts Framework ( Table 8). In seven of the 16 frameworks that included concepts, there was some overlap with the IHC Key Concepts Framework. Of the 13 frameworks that included competences, there was some overlap with the IHC Key Concepts Framework in five. There was very little overlap with the dispositions included in eight frameworks.

### Development of the frameworks

The methods used to develop the frameworks were clearly described for only 10 of the 22 included frameworks, and the basis was clear for only six ( Table 9). In total, 11 of the 22 were based in part on another framework, three on a model or theory, four on a systematic review, nine on an unsystematic review, three on a formal consensus process, and seven on an informal consensus process. The *evidence-based practice core competences* and *Cochrane Risk of Bias Tool* were the most systematically developed frameworks. Both were based in part on systematic and unsystematic reviews. The *evidence-based practice core competences* used a formal consensus process, whereas the *Cochrane Risk of Bias Tool* used an informal process.

**Table 9.** Methods used to develop the frameworks.

Framework	Clear methods	Clear basis	Another framework	Model or theory	Systematic review	Unsystematic review	Formal consensus	Informal consensus	Something else
Critical thinking									
Taxonomy of critical thinking dispositions and abilities	Yes	Somewhat		Dewey's		Yes			
Model of critical thinking	No	No							
List of critical thinking skills	No	No							
Model of the good thinker	No	Somewhat		Dewey's					
Logic and argumentation									
Logical fallacies *	Yes for some	Varies	Various			Yes for some			Logic
Taxonomy of concepts and critical abilities related to the evaluation of verbal arguments	Yes	Yes	Toulmin's						
Evidence based reasoning framework	No	Somewhat	Toulmin's			Yes		Yes	
Cognition									
Cognitive biases *	No	Varies	Various	Various		Yes for some	Yes for some	Yes for some	Qualitative analysis
Framework for understanding people's theories about their own cognition	No	No							
Epistemological models *	Yes	Yes	Various						Interviews & a survey
AIR model of epistemic cognition	No	No							
Scientific thinking									
PISA framework for scientific literacy	No	Somewhat						Yes	International input, feedback, and experience
Framework for K-12 science education	Yes	Yes	Previous U.S. frameworks						An iterative process with input and feedback from partner organisations, design teams, experts, and open public comment
Systems thinking *	No	Varies	Various						Developed over several decades in several different disciplines
Model for scientific thinking	Yes	Yes							A previous study ^143^ and principal components analysis for a measurement instrument
Evidence-based health care									
Health literacy frameworks *	Yes for some	Varies			Yes for some	Yes for some		Yes for some	Concept mapping ^146^
Evidence-based practice (EBP) core competencies	Yes	Yes	5 steps of EBP		Yes	Yes	Yes		
GRADE ^†^	No	Somewhat	Preceding systems for grading evidence and recommendations		Yes	Yes		Yes	Testing criteria against examples. It has gone from an informal consensus process to having explicit decision rules [ https://www. gradeworkinggroup.org/docs/Pub- rules-20170928.pdf]
Bradford-Hill criteria	No	No							
Critical appraisal *	Yes for some	Varies	Preceding checklists			Yes for some	Yes for some	Yes for some	Surveys, pilot validation testing
Cochrane risk of bias tool ^†^	Yes	Yes	Previous checklists		Yes	Yes		Yes	Pilot testing
Catalogue of biases	No	No	Although the catalogue was inspired by David Sackett's list of biases, it is not clear what methods were used to create this list.						
N “yes” or “yes for some” ^‡^	10	6	11	3	4	9	3	7	
Percent	45%	27%	50%	14%	18%	41%	14%	32%	

* More than one framework was considered. ^†^ Although more than one framework was considered, the assessment applies to this specific framework ^‡^ Yes or yes for some for “clear methods”; yes for other bases

### Evaluations of the frameworks

Key findings of formal and informal evaluations of the included frameworks are summarised in Table 10. We found formal evaluations of seven of the 22 included frameworks. Methods used to formally evaluate the frameworks included factor analysis ^143, 146, 187^; extensive feedback (including online surveys) ^32^; principal components and Rasch analysis ^143^; systematic reviews ^24, 29, 157, 175, 181, 185, 188^; an agreement study ^189^ and an assessment of the effect of training on reliability ^182^; and an assessment of usability using focus groups and online surveys ^183^. Two frameworks were evaluated both formally and informally, were found to be useful, and are widely used: the *GRADE framework* ^24, 157, 188, 189^ and the *Cochrane Risk of Bias Tool* ^181– 183, 184, 185^.

**Table 10.** Key findings of formal and informal evaluations of the frameworks.

Name	Formally evaluated	Key findings	Informally evaluated	Findings
Critical thinking				
Taxonomy of critical thinking dispositions and abilities	No		Yes	The underpinning values of Ennis’ work are those of rationality and logical thinking, with little attention paid to the impact of feelings on thinking. Elsewhere, Ennis defends critical thinking against cultural bias, whilst accepting that culture and context have serious implications for such an approach. Ennis aimed to produce a taxonomy which enables critical thinking to be used practically. He says that his taxonomy is ‘simple and comprehensible’ and considers that it can be implemented successfully in different ways, though he acknowledges that it needs further research to validate detailed aspects ^12^.
Model of critical thinking	No		Yes	Paul's lists of abilities and traits do not have any significant omissions when compared with those of Ennis or Perkins, Jay and Tishman. Nosich believes that it is because Paul’s model of critical thinking is concept- based (as opposed to having rules, procedures or steps to follow), that it is effective in curriculum development. The model is extremely flexible, applicable to any subject matter and to any level of thinking. Paul’s major contribution to the area of critical thinking is his idea of ‘weak’ versus ‘strong sense’ critical thinking. The latter is what Paul refers to as the ability to discover and contest one’s own egocentric and socio-centric habits of thought. Paul claims that his nine traits of thought, which are moral commitments and intellectual virtues, transfer thinking from ‘a selfish, narrow-minded foundation to a broad open-minded foundation’ ^12^.
List of critical thinking skills	No		Yes	Halpern does not claim to have provided comprehensive lists of critical thinking skills. It is possible to identify many gaps in her lists. More than any author whose work we have reviewed, Halpern has endeavoured to translate theory and research from cognitive psychology into a form where it can be useful in everyday life. There is up-to-date teaching material to accompany the main text. She has also drawn on relevant sources outside psychology. Halpern is a strong believer in the application of rational methods in problem-solving, including the use of controlled experiments. She points to the need for people to learn how to learn and to be critically selective in responding to the barrage of information (including advertisements and political rhetoric) around them. She argues that teaching and assessing critical thinking will improve the quality of teaching and learning at college level and will increase social capital and economic competitiveness. These are pragmatic arguments, in support of which she cites several studies to illustrate the transferability of critical thinking skills ^12^.
Model of the good thinker	No		Yes	When compared with other critical thinking theorists, there are some serious gaps in their enumeration of the qualities of a good thinker. Empathy, humility, respect for other points of view, clarity and integrity are signally absent. It is also surprising that building understanding, justification, seeking consensus, and formal problem-solving are not included in the list of ‘common’ types of thinking. The list of the general characteristics of the good thinker is of limited value in determining what counts as the rational pursuit of goals in a situation. It is almost a truism that irrational, impulsive, rigid, restricted, self-satisfied and biased thinking are to be avoided. Baron provides clear definitions and examples from diverse domains, including real-life problems. His model is easy for teachers and learners to understand, but the most valuable part of it is the simplest: the idea of thinking and learning as enquiry ^12^.
Logic and argumentation				
Logical fallacies	No		Yes	There does not appear to be agreement on a framework.
Taxonomy of concepts and critical abilities related to the evaluation of verbal arguments	Yes	A factor analytic study of the Wisconsin Tests of Testimony and Reasoning Assessment (WISTTRA) ^187^: Subject matter specialists in speech developed a taxonomy of concepts and abilities related to verbal argument as used in ordinary discourse. It was the purpose of this study to use data collected to assess these hypothesized abilities to determine, using factor analytic procedures, the construct validity of the taxonomy. Both derived orthogonal and derived oblique factor solutions were obtained for each of three initial factor methods. The major conclusion was that the tests based upon the taxonomy have construct validity at a level of specificity.	Yes	This is a distinctive model in that brings together in an economical form a set of concepts and abilities which can be used in many content areas. The scope of the model is rather narrow, covering only a subset of the 15 critical thinking abilities identified by Ennis ^12^.
Evidence based reasoning framework	No		Yes	It may be useful as a framework for assessment - evaluation of the quality of arguments ^87^.
Cognition and epistemology				
Cognitive biases	No		Yes	A systematic review of the literature revealed a total of 76 differently named decision biases or sources of decision biases. An examination of the biases suggests that there are several similarities, and possible overlap, among many of the biases, despite being assigned different names by different researchers. While some researchers have attempted to create classification schemes of decision biases, all the existing categorizations are based on subjective groupings, and none are mutually exclusive and exhaustive. To support further research in the fields of economics, psychology, and managerial decision-making, and to more effectively introduce these biases to the supply management discipline, the authors developed a taxonomy of these decision biases using a systematic, scientifically valid methodology which results in a classification which is both mutually exclusive and exhaustive. The authors are unaware of any other research which has used a scientifically valid set of methodologies to develop a mutually exclusive and exhaustive taxonomy of decision biases ^190^.
Framework for understanding people's theories about their own cognition	No		No	
Epistemological models	No		Yes	There were numerous limitations to Perry’s original study. The scheme's lower positions are more explicitly epistemological than the upper positions, which shift "away from spatial-cognitive restructuring to emotional and aesthetic assessments". Thus, while the epistemological movement from dualism to relativism is clearly noted, how knowledge is construed beyond these positions is less well defined. Perry's work came under attack in the late 1970s for the limitations of generalizing from an elite male sample to the general population of college students. One of the persistent difficulties faced by those who wished to utilize the scheme as more than a theoretical lens has been the difficulty in operationalizing the scheme and in measuring change. Perry did not conduct further research to explore linkages between his conception of epistemological development and student learning, but he did speculate in later work on possible connections among cognitive styles, learning strategies, and development. "When students radically revise their notions of knowledge, would they not be likely to change their ways of going about getting it?" Baxter Magolda attempted to explore gender-related patterns of epistemological development by studying both men and women conducted a longitudinal study of college students at one institution. Epistemology, as it appears to have been defined in this study, largely consisted of student perceptions of learning experiences. It may be problematic that actual reflective judgment, noted in Stages 6 and 7 of King and Kitchener’s framework appears to have been attained by only a minute fraction of those interviewed and has appeared consistently only among advanced graduate students. Responses to the hypothetical problems posed in the interviews may tell us little about how student beliefs are aroused in actual experiences. We know little about how reflective judgment develops in context and just how education makes a difference Kuhn appears to use a simplified three-stage representation of Perry's scheme and offers little information as to the empirical validation of this scheme, but in the connection of epìstemological theories to reasoning. Kuhn's work seems least clear in the definition of elements that comprise epìstemological theories. Conceptually, the theoretical rationale for the four dimensions in Schommer’s framework is somewhat problematic. Measuring epistemological beliefs in paper-and-pencil questionnaire format is an attractive and expedient alternative to interviews. However, considerable questions remain about this approach, as well as about this particular use of survey methodology. There are several important conceptual and methodological issues to be resolved in future research. We believe that one of the most important issues is the definition and delineation of the construct of epistemological beliefs and thinking ^42^.
AIR model of epistemic cognition	No		No	
Scientific thinking				
PISA framework for scientific literacy	No		Yes	The framework was reviewed by expert panels in each of the participating countries, but their feedback was not reported ^123^. “Procedural and epistemic knowledge are very important for interpreting claims from researchers, for instance in media reports. Just content knowledge is not sufficient to understand how science works. However, the description of knowledge is fairly theoretical and might be read as a syllabus for a course on the philosophy of science. I am convinced that this is not the intention, so the main challenge for PISA 2015 is to write test items which are feasible for 15-year olds at various ability levels” ^191^. “The OECD sets out to determine scientific literacy for future adult life through a longitudinal international study, although this has been criticised, not least because its measures are through written tests and questionnaires, which generally show developing countries to be in poor shape to meet such a goal.” “In this paper it is suggested that retaining the use of scientific literacy is still appropriate, but it is necessary to relate scientific literacy to an appreciation of the nature of science, personal learning attributes including attitudes and also to the development of social values. For this, relevance of the learning plays a role and teaching materials, striving toward student enhancement of scientific literacy, need to consider a societal frame, introduction of conceptual science on a need to know basis, and to embrace the socioscientific situation that provides the relevance for responsible citizenship.” “The trend in defining scientific literacy is suggested as away from the short term product approach, in which the facts and skills are paramount, towards the inclusion of issue- based teaching, the need to go beyond scientific problem solving to encompass socioscientific decision making, and the recognition that scientific literacy relates to enabling citizens to effectively participate in the real world. The trend indicates a movement that gives less attention to scientific literacy being viewed as the possession of conceptual understanding of pure science abstract ideas and emphasises more the ability to make decisions related to the technological applications of scientific ideas or socioscientific issues facing society, these being recognized as crucial learning components” ^121^. There is a widespread critique of many aspects of PISA in academic articles, and from many different disciplines ^192^.
Framework for K-12 science education	Yes	There was extensive feedback on a draft ^32^. In general, the feedback about the draft framework indicated support for the overall approach. In the online surveys, many individuals commented that they were impressed with the document and thought it provided a good next step toward refining standards for K-12 science education. At the same time, there were many critiques and suggestions for how to improve it. In looking across all the modes of gathering feedback, some key overarching issues emerged: • concerns about the purpose, audience, and voice; • suggestions of additional fields or topics to include; • how best to incorporate and describe ideas in engineering and technology; • concerns that there was too much material; • lack of guidance or examples about how to convey the integration of crosscutting concepts, core ideas, and practices; • insufficient indication of connections to other topics or issues, such as mathematics and literacy; • need for a stronger statement about science for all and insufficient attention to diversity and equity; • lack of “standards” for curriculum, programs, assessment, and professional development similar to those that were included in the National Science Education Standards; and • lack of attention to the challenges inherent in implementing the framework.	No	
Systems thinking	No		Yes	See above
Model for scientific thinking	Yes	A measurement tool based on the model was evaluated using principal components analysis, the Rasch model, and confirmatory factor analysis. There was good fit for the “Scale for Scientific Thinking”. “The factors confirmed for scientific thinking and self-regulation in conducting research together with creativity were structured in a measurement model to test if they are related. It was previously hypothesized that the constructs scientific thinking, self-regulation in research, and creativity converge with each other. Their point of convergence was primarily explained in the social cognitive theory and field theory. The hypothesis was supported in the present study. The constructs scientific thinking, self- regulation in research, and creativity were significantly correlated in the measurement model” ^143^.	No	
Evidence-based health care				
Health literacy frameworks	Yes	On the basis of questionnaire data, a “quantitative” structural model was created by first applying exploratory factor analyses and then cross-validating the model with confirmatory factor analyses. “The questionnaire proved to be reliable and valid, and the structural model was replicated and cross-validated via structural equation modelling with different samples.” “The model presented here adds to the picture of health literacy derived empirically by Jordan *et al.* which also relied on concept mapping. In that study, Jordan developed the construct of health literacy from a patient perspective. While the patient perspective is important, Jordan *et al.* stated, “in addressing health literacy the focus should not lie solely with the patient.” Our study addresses this point by expanding the perspective on health literacy with input from experts in healthcare and demonstrates where the different perspectives (patient vs. provider) share similar ideas about health literacy” ^146^.	Yes	There is not a consensus on a definition or a model.
EBP core competencies	No		No	
GRADE and related frameworks	Yes	There have been comparisons to other systems and agreement studies. All the approaches used to grade levels of evidence and the strength of recommendations prior to GRADE had important shortcomings ^188^. A review of systems for rating the quality (certainty) of evidence found that GRADE was unique in its comprehensive guidance, rigorous development, and dissemination strategy ^157^. A review of decision-making frameworks for coverage decisions ^24^ found that: “Although no modifications to the GRADE EtD framework for coverage decisions appeared necessary to address the situation of effective but expensive and desirable interventions, modifications to some parts of the seven-construct framework - burden of disease, benefits and harms, values and preferences, resource implications, equity, acceptability, and feasibility - would increase its applicability in a range of political and health systems. Suggested modifications to the GRADE EtD framework include adding the consideration of limitations of the alternative technologies in use (as an elaboration of benefits and harms) and, more importantly, broadening acceptability and feasibility constructs to include political and health system factors. Findings of an agreement study suggest that trained individuals using the GRADE approach improves reliability in comparison to intuitive judgments about the QoE and that two individual raters can reliably assess the QoE using the GRADE system ^189^.	Yes	Although there have been criticisms of the GRADE approach, it is now the most widely used and highly cited approach. [ https://www. gradeworkinggroup.org/]
Bradford Hill criteria	No		Yes	The framework does not reflect the current, more clearly articulated view of causal processes. Additionally, the guidelines used to evaluate evidence have not changed for decades, even as the causal questions have become more complex, beyond the original intent of this framework. One important limitation of the classic view of disease causation arising from the Hill criteria has been the lack of a formal basis for evaluating causal hypotheses. Only in the past several decades have investigators explored more formally the foundational mathematical and conceptual issues required for rigorous estimation of causal effects, particularly in circumstances where randomization of treatment assignment that ensures exchangeable comparison groups is unfeasible. The inference about cause became the rationale for intervention, but the causal conclusions were not couched in the consequences of specific actions to reduce or eliminate cigarette smoking. And later, public health action was aimed at the individual smoker, rather than at the upstream system of cigarette manufacture, advertising, and distribution. This limited focus is a key characteristic of the traditional approach; causal determinations were made by epidemiologists and others in public health about various risk factors without considering the effect of a specific way of changing them. The utility of long-used, familiar approaches for statistical analysis and causal inference to interpret the broad sweep of evidence on the causal determinants of human health is diminishing. Public health practitioners and researchers must understand the limitations of those ^162^. They have to some extent withstood the test of time, in that they are still widely recognised and taught, but they are increasingly being replaced by GRADE (and other frameworks) and they are, in some ways, not consistent with GRADE ^165^.
Critical appraisal	Yes	Katrak ^29^ reported a systematic review of critical appraisal tools. Many published critical appraisal tools are available to critically appraise research reports. Many of the tools were reported to be modifications of other published tools or reflected specialty concerns in specific clinical or research areas, without attempts to justify inclusion criteria. Few of the generic critical appraisal tools could be usefully applied to any health research. Forty-two different items were extracted from the six critical appraisal tools that could be used to evaluate randomised and non- randomised studies. The majority of the critical appraisal tools were developed for a specific research design (87%), with most designed for use on randomised studies (38%). There is also a considerable number of critical appraisal tools for systematic reviews (N = 26). There is a lack of information on tool development processes in most cases. Only 14 out of 121 instruments (12%) were reported as having been constructed using a specified empirical approach. Few critical appraisal tools had documented evidence of validity of their items, or reliability of use. Face validity was established in nine critical appraisal tools, seven of which were developed for use on experimental studies and two for systematic reviews. Intra-rater reliability was established for only one critical appraisal tool as part of its empirical development process, whereas inter-rater reliability was reported for two systematic review tools (for one of these as part of the developmental process) and seven experimental critical appraisal tools (for two of these as part of the developmental process). Hyde ^175^ reported a systematic review of evaluations of the effects of critical appraisal workshops, most of which use checklists. Sixteen studies met the inclusion criteria. One study was an RCT, 8 were non-randomised between group studies, and 7 were before-and-after studies. The impact of critical appraisal teaching on clinicians’ behaviour (principally reading behaviour) was mixed. Of the eight comparisons for this outcome six had major threats to validity. Most, but not all, of the comparisons showed benefit of critical appraisal teaching, two acting in the opposite direction. Critical appraisal teaching was seen to consistently increase skills: fourteen of the sixteen comparisons for this outcome showed a positive effect. The strength of the effect remained when self-assessed comparisons were removed. Five comparisons were thought not to be subject to major flaws: four of these indicated a benefit of critical appraisal teaching. The strongest and most consistent impact of critical appraisal teaching was seen on knowledge outcomes: 7 of the 12 studies showed a statistically significant positive effect. However, consideration of the size of the benefit revealed heterogeneity. There were four comparisons of the impact on attitudes - all were positive, but it was not possible to separate out real effects from a tendency for participants to respond in a “desired” manner. There were inadequate data to assess whether there was variation in outcome according to the mode of delivery of the educational intervention.	Yes	There is variation among available critical appraisal tools.
Risk of bias	Yes	West *et al.* ^185^ and Bai *et al.* ^181^ assessed whether instruments considered all or most of the elements for each domain and did not omit any element defined as essential. Savovic *et al.* ^183^ assessed the usability of the Cochrane risk of bias tool by means of focus groups, online surveys and a face.to- face meeting. Da Costa *et al.* ^182^ assessed whether intensive, standardized training on risk of bias assessment improved the reliability of the Cochrane risk of bias tool. The Cochrane RoB tool was experienced positively by users and its reliability could be improved by training. There appears to be broad agreement regarding the criteria included in the Cochrane RoB tools.	Yes	Zeng *et al.* ^184^ considered the Cochrane RoB tool the best available tool for randomised trials. Viswanathan *et al.* ^180^ provided general guidance without recommending a specific tool.
Catalogue of biases	No		No	

Our assessment of the elements (concepts, competences or dispositions) in the 22 frameworks is summarised in Table 11. Only one framework, the *framework for K-12 science education,* had clear inclusion criteria for one of three dimensions (“core ideas”). We judged the elements to be coherent in five frameworks, distinct in nine, and organised logically in eight. There were no inappropriate elements in seven frameworks and no missing elements in two. Overall, the *evidence-based reasoning framework* ^86^ was the only framework that we assessed positively for all five criteria (coherent elements, distinct elements, no inappropriate elements, no missing elements, and logical grouping of the elements). That framework is a relatively simple analytic model of arguments about scientific ideas.

**Table 11.** Assessment of the frameworks.

Framework	Clear inclusion criteria	Coherent elements ^§^	Distinct elements **	Inappropriate elements ^††^	Missing elements ^‡‡^	Logical grouping ^§§^
Critical thinking						
Taxonomy of critical thinking dispositions and abilities	No	Somewhat	Somewhat	Possibly	Possibly	Possibly
Model of critical thinking	No	Yes	Yes	Possibly	Possibly	Possibly
List of critical thinking skills	No	No	Somewhat	Possibly	Possibly	Possibly
Model of the good thinker	No	Not clear	No (except for dispositions)	No	Yes	Possibly
Logic and argumentation						
Logical fallacies *	No	Varies	No	Possibly	Possibly	Yes (although the logic that is used varies)
Taxonomy of concepts and critical abilities related to the evaluation of verbal arguments	No	No	Yes	No	Yes	Yes
Evidence based reasoning framework	No	Yes	Yes	No	No	Yes
Cognition						
Cognitive biases *	Varies	Varies	No	Possibly	Possibly	Possibly (although the logic that is used varies)
Framework for understanding people's theories about their own cognition	No	No	Yes	Not clear	Possibly	Possibly
Epistemological models	No	Somewhat	Somewhat	No	No	Yes
AIR model of epistemic cognition	No	No	Yes	No	Yes	Yes
Scientific thinking						
PISA framework for scientific literacy	No	No	Somewhat	No	Possibly	Possibly
Framework for K-12 science education	For one dimension (core ideas) only	Yes within each dimension, not across dimensions	Yes	Possibly	Yes	Not clear
Systems thinking *	No	Somewhat	Somewhat	Possibly	Possibly	Possibly
Model for scientific thinking	Based on principle components analysis	Yes	Yes	Possibly	Possibly	Possibly
Evidence-based health care						
Health literacy frameworks *	No	Varies	Yes (within different models)	Possibly	Possibly	Possibly
Evidence-based practice (EBP) core competencies	There was a predefined consensus level (70%), but no explicit criteria for the people making judgements.	Somewhat	Somewhat	Possibly	Possibly	Yes
GRADE ^†^	No	Somewhat	Somewhat	Possibly	Possibly	Yes
Bradford-Hill criteria	No	Somewhat	Somewhat	Yes	Yes	Possibly
Critical appraisal *	No	Varies	Possibly (within checklists), not across checklists	Varies	Possibly	Possibly
Cochrane risk of bias tool ^†^	No	Yes	Yes	No	Possibly	Yes
Catalogue of biases	No	No	No	Yes	Possibly	No
N frameworks "yes" ‡	1 (partially)	5	9	7 (no)	2 (no)	8
Percent	5%	23%	41%	32%	9%	36%

* More than one framework was considered. ^†^ Although more than one framework was considered, the assessment applies to this specific framework ^‡^ Yes or yes for some for “clear methods”; yes for “coherence”, “distinct”, and “logical grouping”; no for “inappropriate elements” and “missing elements” ^§^ Does not mix type(s) and specificity of concepts, competencies, or dispositions ^** ^Included concepts, competencies, or dispositions are clearly different from each other ^††^ Concepts, competencies, or dispositions included in the framework that should not have been ^‡‡^ Concepts, competencies, or dispositions not included in the framework that should have been ^§§^ Concepts, competencies, or dispositions organised in a way that makes sense

### Use of the frameworks

Information about how the 22 frameworks have been used is summarised in *Extended data File 4*. We found evidence that most of the frameworks were being used. For four (the *taxonomy of concepts and critical abilities related to the evaluation of verbal arguments*, the *evidence-based reasoning framework*, the *AIR model of epistemic cognition*, and the *model for scientific thinking*) we found little evidence of use. Two had only been available for one or two years (the *evidence-based practice core competences* and the C *atalogue of Biases*), and we were uncertain about their use. Twelve of the frameworks appeared to be intended primarily for teachers and students, and we found learning resources based on 14 of the frameworks.

Nine of the frameworks appeared to be intended primarily for researchers. One (the *evidence-based practice core competences*) appeared to be intended primarily for curriculum developers ^26^. We found at least some evidence that six other frameworks were used for curriculum development, including three of the critical thinking frameworks. We found evidence that 12 of the frameworks were used as the basis for one or more assessment tools. Other ways in which the frameworks have been used or have been proposed for use include: self-teaching; by parents, institutions, and government; by employers developing training programs; professional development; establishing norms or standards; developing ways of protecting against cognitive biases; theory development; intervention design; policy advice; and reporting standards.

### Strengths and weaknesses of the frameworks

Strengths and weaknesses of each framework and ideas for further development of the IHC Key Concepts Framework are summarised in Table 12. Strengths of the frameworks related to their development include international collaboration, support from international or national organisations, continued development over a long period of time, well described and systematic development, research evidence to support all of the concepts, elicitation of extensive feedback, and formal comparisons to similar frameworks. Strengths related to their usability include simplicity, a user-friendly structure for describing each concept, and wide use.

**Table 12.** Strengths, weaknesses, and ideas for IHC Key Concepts.

Framework	Strengths	Weaknesses	Ideas for IHC Key Concepts
Critical thinking			
Taxonomy of critical thinking dispositions and abilities	Continued development over more than 40 years.	No formal evaluation. It has a broad focus and may be difficult to apply to thinking critically about health claims and choices or other specific decisions about what to believe or do.	Trace the origins of the IHC Key Concepts back to clinical epidemiology, critical appraisal, and evidence-based practice. Ennis analyses different approaches to assessing critical thinking, rejecting multiple-choice assessment for all but self-assessment and research. He also questions performance-based assessment on grounds of cost, focus and context (the more realistic the performance the more complex the problem). “Reasonable reflective thinking focused on deciding what to believe or do” is a good way of describing the aim of the IHC Key Concepts. Consider using a figure or model for organising the concepts
Model of critical thinking	Establishment of a centre, scholars, and an annual conference ( www.criticalthinking.org). Outline of a spiral curriculum ^193^	Unclear development methods No formal evaluation	IHC annual conference, fellows, guides, online courses, library Clarification of what is outside of the IHC scope, including “fair mindedness” / ethics Consider different audiences. Consider the extent to which IHC approach may conflict with Paul approach.
List of critical thinking skills	Used and refinement based on feedback between 1984 and 2014. Translation of cognitive psychology theory and research into a form where it can be useful in everyday life. Halpern is a strong believer in the application of rational methods in problem-solving, including the use of controlled experiments.	Unclear development methods Developed by a single author with a background in cognitive psychology No formal evaluation	Seek feedback from people with a strong background in critical thinking Consider reframing concepts as skills and adding examples of use. Consider developing a textbook for older students and teachers.
Model of the good thinker	The purpose of rational (critical) thinking is explained nicely, the basic framework is simple and logical, focusing on thinking and learning as enquiry, and there are some useful definitions. The textbook introduces relevant theories and approaches to critical thinking, and uses examples from diverse domains, including real-life problems.	Unclear development methods No formal evaluation There is not a clear list of concepts or competences that should be learned and very little about how they should be learned or evaluated.	Consider adapting some of the text and definitions used to describe good thinking.
Logic and argumentation			
Logical fallacies	They are based on logic and are useful for analysing arguments and recognising logical fallacies.	No formal evaluation There is more than one logic that can be used to organise logical fallacies. None of the frameworks (lists) seem to have been developed systematically for teaching and learning, and the ones that are for teaching and learning seem more ad hoc, without an obvious underlying logic. Many lists are long, and it is not clear how they are learned and remembered. Lack of an appropriately selected, labelled, explained and organised list that is optimised for teaching/learning, remembering and using	Systematically consider all the logical fallacies and clarify why some logical fallacies are excluded from the Key Concepts. Consider the logic we have used to organise the IHC Key Concepts and ways of making the Key Concepts easier to learn and remember. Explore how a logician would approach evaluating the IHC framework. Clarify similarities and differences between the Key Concepts and logical fallacies Consider lessons from the School of Thought website, cards, and poster, which have gone viral.
Taxonomy of concepts and critical abilities related to the evaluation of verbal arguments	Broad focus on a framework that can be applied to any type of argument (including causal claims) Builds on a simple model for arguments	Because the framework is so broad, it is inadequate as a framework for assessing claims about effects. Assumption that “The layman cannot, under normal circumstances, verify (in any rigorous sense) the technical information he must use.” The 5th critical ability (“Recognizing testimony offered as justification”) and 6th (“Appraising testimony in terms of internal and external criteria”) are based on the authority of the person offering testimony.	Develop a model that organises the Key Concepts logically Determine whether the difference between ‘critical abilities’ (skills or proficiencies) and competence (the required skill, knowledge, or capacity to do something) is important and whether IHC should specify one or the other or both, if there is an important distinction
Evidence based reasoning framework	Collaboration of an international consortium that piloted using the framework for assessments of scientific reasoning It is a simple model	Unclear development methods No concepts or criteria for assessing the trustworthiness of evidence or claims, or for going from claims to decisions, and it is unclear how the model could be used as a starting point for concepts, competences, or dispositions other than as a possible way of organising these. It appears to be like teaching sentence structure. While it might be useful for analysis, it is not obvious how useful it is for teaching and learning. No formal evaluation	International collaboration in further development Consider assessing the impact of using IHC learning-resources on argumentation, using a tool based on this framework or other tools. Consider using the IHC Concepts as a framework for making claims as well as for assessing claims made by others. Consider whether there is a simple model or figure that could be used to illustrate the Key Concepts
Cognition			
Cognitive biases	Strategies for being aware of and protecting against cognitive biases All the biases are based on research evidence.	Unclear development methods There does not seem to be agreement or any attempt to reach a consensus or develop an optimal framework for cognitive biases for teaching and learning. Several frameworks are long lists without a widely appealing way of grouping, remembering and using the concepts. No formal evaluation	Incorporate evidence for relevant cognitive biases in systematic summaries of the evidence for each IHC Key Concept A popular book like Kahneman’s ^92^, poster and website like School of Thought ^94^, and a Wikipedia page Address why some cognitive biases are included, and others are not, and the relation of Key Concepts to cognitive biases.
Framework for understanding people's theories about their own cognition		Unclear development methods No formal evaluation	Consider developing a formal metacognitive theory. Consider implications for teaching the IHC Key Concepts: “We believe that schools should actively promote metacognitive theorizing among all students. Research indicates that theorizing improves both performance and understanding of one's performance. Research further supports the claim that metacognitive theorizing can be facilitated by self-talk and peer interactions that focus on the process rather than the product of learning.” Consider incorporating discussion of tacit theories about how students decide what to believe and what to do, helping them to become aware of their tacit theories and to develop formal theories: “Perhaps the most salient aspect of a tacit metacognitive theory as opposed to an explicit one is that an individual is not readily aware of either the theory itself or evidence that supports or refutes it. Thus, tacit theories are not readily distinguished from, or tested against, relevant data. To the extent that they remain tacit, metacognitive theories may be persistent even when they are false and maladaptive.” “One potential advantage of a formal metacognitive theory is that it allows the individual to make informed choices about self-regulatory behaviors.” ^103^
Epistemological models	"Kuhn's contribution to the literature on epìstemological understanding has been in the connection of epìstemological theories to reasoning. The skills of argument appear predicated on a level of epìstemological understanding that requires contemplation, evaluation, and judgment of alternative theories and evidence." It "is notable in its focus on ill-structured problems from everyday life and in the use of a broad sample of participants across the life span. This sampling of a broader population on non-academic issues removes epistemological beliefs from the realm of the classroom and separates issues of knowing from those of teaching and learning processes ^42^.	No formal evaluation "We know little about how reflective judgment develops in context and just how education makes a difference." ^42^	Consider epistemological development in designing a spiral curriculum, deciding the order and when Key Concepts should be taught, and how. Consider organising the Key Concepts in relation to stages of development + exercises designed to help students move from one stage to the next. Also, consider the use of ill-structured problems (problems about which reasonable people reasonably disagree) ^111^. “In concluding, it is worth noting that the timing of educational efforts may have important consequences.” ^43^
AIR model of epistemic cognition	Potentially useful for informing the design of interventions	Largely theoretical at this time, with little practical use There is very little description of how the model was developed. No formal evaluation	Consider implications for addressing when and how people should assess claims and evidence and take the time to analyse a decision, when they should ignore claims or evidence, and when they should rely on others.
Scientific thinking			
PISA framework for scientific literacy	International development and input, informed by triennial testing of 15-year-old students in many different countries	Development of the framework is poorly described No formal evaluation	International input International comparisons - Compare how well students do on PISA science to how well they do on a Claim test. Consider using PISA to measure impact of IHC school resources on scientific literacy.
Framework for K-12 science education	Extensive feedback + a summary of the feedback and responses ^32^	Too little focus on applied science, practical understanding and use of science by non-scientists, and what children will remember and make use of. "In the K-12 context, science is generally taken to mean the traditional natural sciences: physics, chemistry, biology, and (more recently) earth, space, and environmental sciences. In this document, we include core ideas for these disciplinary areas, but not for all areas of science” ^32^. Although it appears to be having a big impact in the U.S., it is unclear whether it is having much of an impact outside the U.S. This may reflect a downside of the way in which it was developed without international engagement.	More extensive feedback, including feedback from relevant organisations and focus groups Clarify how our focus fits with broader science framework, need for focus on using research and making informed choices versus doing research. Clarify important goals for science education that are outside of the IHC scope. Consider goals.
Systems thinking		Unclear development methods Lack of systematic development of any framework for systems thinking, so far as we are aware No formal evaluation	May be relevant for assessments of models when they are used to evaluate interventions, specifically models that are based on systems thinking Clarify the application of the Key Concepts to system interventions
Model for scientific thinking	Consideration of how scientific thinking, self-regulation in research, and creativity relate to each other	Focus on traits or behaviours, not on competencies or concepts. Not used for teaching and unclear implications for teaching	
Evidence-based health care			
Health literacy frameworks	Systematic reviews Support of international and national organisations such as the World Health Organization and the U.S. National Academy of Sciences Epidemiological studies showing association between health literacy and health outcomes	More attention appears to have been given to measurement than to intervention development and evaluation ^194^.	Consider framing purpose of IHC Key Concepts in terms of critical health literacy. Clarify why we have excluded obtaining and understanding information. Consider developing a model that includes moderators and mediators, building on process evaluations. Consider Rasch analysis as a way of validating IHC Key Concepts framework.
Evidence-based practice (EBP) core competencies	Well described, systematic development methods	The broad approach across professions might limit the relevance to specific professions. “Although we selected Delphi participants to represent a diverse range of health professionals and expertise, they may not adequately represent the full spectrum of views held by individuals within a single profession” ^26^. No formal evaluation	
GRADE and related frameworks	Consensus developed over a long period of time involving people who were responsible for developing and using other systems, open meetings, wide use, and ongoing development Formal comparison to other approaches, study of agreement among people applying the approach It could potentially be used as a framework for helping people to make judgements about the trustworthiness of evidence and for making informed choices.	Development of the framework is not well described GRADE is often experienced as complex.	Involvement of a diverse group of people with relevant experience and expertise over a long time, with regular meetings Application of the framework to multiple examples (of claims, comparisons, and choices) that challenge our thinking Consider the extent to which GRADE criteria should be incorporated that may not clearly be within the scope of the Key Concepts. Consider the pros and cons of how the Key Concepts are organised in relation to GRADE criteria. Training workshops on using the Key Concepts Identify target audiences for the Key Concepts, engage them, and make the Key Concepts more accessible and useful to them.
Bradford-Hill criteria	Has provided a useful structure for reviewing evidence, such as evidence of the harmful effects of smoking Widely used	There is almost no description of how the framework was developed. No formal evaluation “The framework does not reflect the current, more clearly articulated view of causal processes. Additionally, the guidelines used to evaluate evidence have not changed for decades, even as the causal questions have become more complex, beyond the original intent of this framework” ^162^.	Consider creating subsets of Key Concepts for specific purposes; e.g. risk of bias, certainty of the evidence.
Critical appraisal	Checklists have been widely used as teaching tools in critical appraisal and evidence-based practice workshops and have evolved based on experience with their use ^195^.	Lack of explicit selection criteria and, for the most part, little if any description of how most of the checklists (particularly those that are intended to be teaching tools) were developed ^29^.	Systematically review checklists designed as teaching tools or decision aids. Use of subsets of Key Concepts as a framework for developing tools such as checklists for specific purposes; e.g. assessing claims or figuring out what to do. Consider evaluating the Key Concepts as a framework for developing learning resources.
Cochrane risk of bias tool	Well described, systematic development and formal evaluation		Use of the Key Concepts related to the risk of bias to assess the overall risk of bias Assess usability and reliability of subsets of IHC Key Concepts
Catalogue of biases	The structure for each entry (background, example, impact, preventive steps)	There is little transparency regarding how the list was developed and it does not appear to have been developed systematically.	Use some of the references as evidence to support relevant Key Concepts. Consider a more structured presentation of each concept, drawing on the headings used in the catalogue. Consider a more user-friendly presentation of the Key Concepts (e.g. with a summary table (something like the Nature table) and one page for each concept, instead of the current table, which people find overwhelming.

Weaknesses of the frameworks include unclear development methods, lack of formal evaluation, multiple frameworks with the same focus and no apparent agreement or effort to reach a consensus on an optimal framework, and complexity or many included concepts or competences.

### Ideas for further development of the IHC Key Concepts Framework

We identified several ways in which the IHC Key Concepts Framework might potentially be improved ( Table 12). These include making the evidence that supports each IHC Key Concept explicit, including evidence of the extent to which each IHC Key Concept is not widely understood or applied; designing a website to popularise teaching and learning about, understanding of, and application of the IHC Key Concepts Framework; and developing a visual model of the IHC Key Concepts Framework.

Overall, our review of the concepts, competences, and dispositions in the 22 frameworks led us to add four new concepts to the IHC Key Concepts Framework, to modify 16, and to add 10 new competences and four new dispositions ^4^.

## Discussion

We identified 22 frameworks that overlap with the IHC Key Concepts Framework. We found that the purpose of the IHC Key Concepts Framework is most like two frameworks for critical thinking: Ennis’ *taxonomy of critical thinking dispositions and abilities* and Baron’s *model of the good thinker*. However, in terms of concepts and competences, there was more overlap with Halpern’s *list of critical thinking skills*. Although the IHC framework drew on evidence-based health care frameworks, there was at most some similarity with the purposes of those frameworks and the purpose of the IHC Key Concepts Framework. There was some overlap in terms of concepts with GRADE, critical appraisal tools, and the *Catalogue of Bias*. There was overlap in terms of competences with health literacy, the *evidence-based practice core competences*, and critical appraisal tools.

We found the IHC Key Concepts Framework to be central to critical thinking and evidence-based practice, both of which have broader scopes than the IHC Key Concepts Framework. An important weakness we found with these and other broad frameworks, such as those that focus on argumentation, is that they do not provide an adequate basis (concepts) for thinking critically about claims about the effects of interventions and decisions about what to do. As noted by Dewey: “It would be impossible to over-estimate the educational importance of arriving at conceptions: that is, meanings that are general because applicable in a great variety of different instances in spite of their difference. They are known points of reference by which we get our bearings when we are plunged into the strange and unknown. Without this conceptualizing, nothing is gained that can be carried over to the better understanding of new experiences” ^196^. The IHC Key Concepts are applicable to a great variety of claims about the effects of interventions, not just health interventions ^7^, and they are essential points of reference for deciding which claims to believe and what to do.

We did not find any overlap between the IHC Key Concepts and those included in the *framework for K-12 science education,* and little overlap in the competences. That framework places little focus on applied science, practical understanding and use of science by non-scientists, and what children will remember and make use of in their daily lives. This may be the case for many national science curricula.

Our review has helped us to clarify the goal of the IHC Key Concepts Framework and led us to add four new concepts, 10 new competences, and four new dispositions. In addition, we have identified ways in which we can improve the methods we use to further develop and evaluate the IHC Key Concepts Framework and make it more useful.

Previous systematic and unsystematic reviews have reviewed different types of frameworks with similar purposes, including frameworks for cognitive biases ^190^, epistemic cognition ^42^, health literacy ^22^, assessments of the certainty of evidence and recommendations or decisions ^24, 157, 193^, causal inference ^162^, critical appraisal ^29^, and assessment of the risk of bias ^181, 185^. Moseley and colleagues ^12^ conducted a comprehensive review of frameworks for thinking, which overlaps with and informed our review. However, we are unaware of other reviews with the same scope as this review, whether in terms of the included frameworks or the data that were collected for each included framework.

We used explicit inclusion criteria for frameworks and two review authors independently collected data from included frameworks using a data collection form. Both the eligibility assessments and the data collection required judgement. Although we frequently disagreed, most of our disagreements were minor and all our disagreements were easily resolved. We did not conduct an exhaustive search for relevant frameworks. There may be other frameworks that meet our inclusion criteria. It is possible that other frameworks could add to our findings, but unlikely that they would otherwise substantially change the findings of this review.

## Conclusions

As defined by Moseley and colleagues: “Framework is a general term for a structure that provides support” ^12^. We have systematically considered 22 frameworks that are relevant to supporting critical thinking about claims about the effects of interventions (actions), comparisons (evidence used to support those claims), and decisions about what to do. We have found that the IHC Key Concepts Framework is unique and that it can be improved by building on the ways in which other related frameworks have been developed, evaluated, and made useful. Much of what we have found can also inform the development and evaluation of other frameworks.

## Data availability

### Underlying data

All data underlying the results are available as part of the article and no additional source data are required.

### Extended data

Norwegian Centre for Research Data, Enabling Sustainable Public Engagement in Improving Health and Health Equity, https://doi.org/10.18712/NSD-NSD2817-V1 ^197^.

This project contains the following extended data:

- File 1: Search strategy
- File 2: Critical thinking frameworks eligibility form
- File 3: Critical thinking frameworks data collection form
- File 4: Use of the frameworks
- File 5: PRISMA checklist

Data are available under the terms of the Creative Commons Attribution 4.0 International license (CC-BY 4.0).

### Reporting guidelines

The PRISMA checklist for ‘Comparison of the Informed Health Choices Key Concepts Framework to other frameworks relevant to teaching and learning how to think critically about health claims and choices: a systematic review’, https://doi.org/10.18712/NSD-NSD2817-V1 ^197^.

Information about this dataset can be found in English here: http://nsddata.nsd.uib.no/webview/index.jsp?v=2&submode=ddi&study=http%3A%2F%2Fnsddata.nsd.uib.no%2Fobj%2FfStudy%2FNSD2817&mode=documentation
