# Supplementary material for: Comparison of the Informed Health Choices Key Concepts Framework to other frameworks relevant to teaching and learning how to think critically about health claims and choices: a systematic review
Source: F1000Res. 2020 Mar 5;9:164. [Version 1] doi: 10.12688/f1000research.21858.1 (PMC7670481; doi:10.12688/f1000research.21858.1)
Supplement: Peer review history

# Peer review: Reviewer response for version 1

**DOI:** 10.5256/f1000research.24099.r74232

**Published:** 2020-11-30

**Authors:** Julia Lühnen

**Affiliations:** 1 Institute for Health and Nursing Science, Medical Faculty, al Faculty, Martin Luther University Halle-Wittenberg, Halle (Saale), Germany

Thanks to the authors for this important and interesting paper.

 The Informed Health Choices (IHC) project aims to support people to evaluate the trustworthiness of health claims and to make informed choices. The IHC Key Concepts provide a framework for designing curricula, learning resources and evaluation tools. The framework has been updated and extended regularly. It comprises several concepts, competences and dispositions in different groups.

 Objective of this systematic review was to compare the IHC Key Concepts Framework to other frameworks that are relevant to teaching and learning how to think critically about treatment claims, evidence and choices.

 The frameworks were mainly identified from reviews of frameworks and by searching Google Scholar. Twenty-two frameworks were included. Two authors independently extracted information on purposes, definitions of key terms, included elements, methods of development and evaluation, and the way the frameworks were used to develop curricula, learning resources and assessment tools. Strengths and weaknesses of each framework were assessed. The authors described the frameworks in detail and displayed differences and similarities in comparison to the IHC Key Concepts Framework. They concluded that the IHC Key Concepts Framework is unique and that it can be improved by taking account of the ways in which other related frameworks have been developed, evaluated, and made useful. The findings can also be used to improve other frameworks.

 A possible limitation of the manuscript is the exclusively search in Google Scholar. Underlying algorithms are unknown and searches cannot be replicated reliably. The authors themselves stated that they may have missed frameworks. We agree with them that it is unlikely that additional frameworks would substantially change the findings of the review.

Are the rationale for, and objectives of, the Systematic Review clearly stated?

Yes

Is the statistical analysis and its interpretation appropriate?

Not applicable

Are sufficient details of the methods and analysis provided to allow replication by others?

Yes

Are the conclusions drawn adequately supported by the results presented in the review?

Yes

Reviewer Expertise:

Evidence-based health information, informed decision-making, trainings in evidence-based medicine

I confirm that I have read this submission and believe that I have an appropriate level of expertise to confirm that it is of an acceptable scientific standard.

# Peer review: Reviewer response for version 1

**DOI:** 10.5256/f1000research.24099.r73961

**Published:** 2020-11-16

**Authors:** Loai Albarqouni

**Affiliations:** 1 The Institute for Evidence-Based Healthcare, Faculty of Health Sciences and Medicine, Bond University, Gold Coast, Qld, Australia

Thanks for inviting me to review this interesting systematic review. The authors of this article systematically review and compare frameworks relevant to teaching and learning of critical thinking about claims, evidence, and choices. This is a great article that is tackling a very interesting issue related to the concepts of critical thinking.

 The authors elegantly visualised the overlap between IHC frameworks and other frameworks on a group of Venn diagrams and included a list of definitions and terminologies that are very useful. The authors provide a detailed transparent description of their methods and results – very impressive.

 A couple of comments:

1. Authors might consider describing how they collected the data regarding the use of these frameworks as the basis for curricula/teaching & learning/ assessment tools – as this usually goes beyond the framework publications.
2. Authors mentioned that as a result of this work, they made a few modifications/additions to the original IHC concepts – authors might consider describing/justifying these changes.

I would like to congratulate the authors on this great work.

Are the rationale for, and objectives of, the Systematic Review clearly stated?

Yes

Is the statistical analysis and its interpretation appropriate?

Not applicable

Are sufficient details of the methods and analysis provided to allow replication by others?

Yes

Are the conclusions drawn adequately supported by the results presented in the review?

Yes

Reviewer Expertise:

Evidence-based Healthcare

I confirm that I have read this submission and believe that I have an appropriate level of expertise to confirm that it is of an acceptable scientific standard.

# Peer review: Reviewer response for version 1

**DOI:** 10.5256/f1000research.24099.r73960

**Published:** 2020-11-03

**Authors:** Declan Devane

**Affiliations:** 1 Health Research Board - Trials Methodology Research Network, National University of Ireland Galway, Galway, Ireland; 2 School of Nursing and Midwifery, National University of Ireland Galway, Galway, Ireland; 3 Evidence Synthesis Ireland, National University of Ireland Galway, Galway, Ireland; 4 Cochrane Ireland, National University of Ireland Galway, Galway, Ireland

The authors of this paper have done a good job in conducting a systematic review of frameworks that overlap with, or potentially overlap with, the IHC key concepts framework.

 The Informed Health Choices (IHC) Key Concepts Framework may help build an early foundation for thinking critically about health claims and learning to make decisions informed by evidence. It provides a potential means of going beyond the delivery of information, to the development of critical thinking skills. There are however other frameworks that are relevant to thinking critically about treatment claims, comparisons, and choices. In this paper the authors position the theoretical underpinnings of the IHC Key Concepts Framework in existing frameworks within the fields of health literacy and critical thinking.

 Systematic review methods are detailed and appropriate as are process for decision making. The authors did not conduct an exhaustive search. It is possible therefore that additional frameworks may have been excluded. the authors acknowledge this limitation and I agree with them in that it is unlikely that additional frameworks would substantially change the findings of their review.

 Comparisons between the IHC framework and the included frameworks in the review are tabulated clearly and the accompanying figures demonstrating relationships between, for example, critical thinking and the key concepts framework are welcomed. It is refreshing to see that the findings of the review led the authors to revise their framework by adding concepts, competencies and dispositions.

 Although this paper is focused largely on the context of primary and secondary school education, it has learning for use of the IHC framework outside of these contexts and also for the development and evaluation of other frameworks. The paper also offers a useful structure for cross comparisons of frameworks in any setting or context.

Are the rationale for, and objectives of, the Systematic Review clearly stated?

Yes

Is the statistical analysis and its interpretation appropriate?

Yes

Are sufficient details of the methods and analysis provided to allow replication by others?

Yes

Are the conclusions drawn adequately supported by the results presented in the review?

Yes

Reviewer Expertise:

Evidence syntheses, randomised trials, maternity care

I confirm that I have read this submission and believe that I have an appropriate level of expertise to confirm that it is of an acceptable scientific standard.
